# Requirements for Carnitine Shuttle-Mediated Translocation of Mitochondrial Acetyl Moieties to the Yeast Cytosol

**DOI:** 10.1128/mBio.00520-16

**Published:** 2016-05-03

**Authors:** Harmen M. van Rossum, Barbara U. Kozak, Matthijs S. Niemeijer, James C. Dykstra, Marijke A. H. Luttik, Jean-Marc G. Daran, Antonius J. A. van Maris, Jack T. Pronk

**Affiliations:** Department of Biotechnology, Delft University of Technology, Delft, The Netherlands

## Abstract

In many eukaryotes, the carnitine shuttle plays a key role in intracellular transport of acyl moieties. Fatty acid-grown *Saccharomyces cerevisiae* cells employ this shuttle to translocate acetyl units into their mitochondria. Mechanistically, the carnitine shuttle should be reversible, but previous studies indicate that carnitine shuttle-mediated export of mitochondrial acetyl units to the yeast cytosol does not occur *in vivo*. This apparent unidirectionality was investigated by constitutively expressing genes encoding carnitine shuttle-related proteins in an engineered *S. cerevisiae* strain, in which cytosolic acetyl coenzyme A (acetyl-CoA) synthesis could be switched off by omitting lipoic acid from growth media. Laboratory evolution of this strain yielded mutants whose growth on glucose, in the absence of lipoic acid, was l-carnitine dependent, indicating that *in vivo* export of mitochondrial acetyl units to the cytosol occurred via the carnitine shuttle. The mitochondrial pyruvate dehydrogenase complex was identified as the predominant source of acetyl-CoA in the evolved strains. Whole-genome sequencing revealed mutations in genes involved in mitochondrial fatty acid synthesis (*MCT1*), nuclear-mitochondrial communication (*RTG2*), and encoding a carnitine acetyltransferase (*YAT2*). Introduction of these mutations into the nonevolved parental strain enabled l-carnitine-dependent growth on glucose. This study indicates intramitochondrial acetyl-CoA concentration and constitutive expression of carnitine shuttle genes as key factors in enabling *in vivo* export of mitochondrial acetyl units via the carnitine shuttle.

## IMPORTANCE

This study demonstrates, for the first time, that *Saccharomyces cerevisiae* can be engineered to employ the carnitine shuttle for export of acetyl moieties from the mitochondria and, thereby, to act as the sole source of cytosolic acetyl-CoA. Further optimization of this ATP-independent mechanism for cytosolic acetyl-CoA provision can contribute to efficient, yeast-based production of industrially relevant compounds derived from this precursor. The strains constructed in this study, whose growth on glucose depends on a functional carnitine shuttle, provide valuable models for further functional analysis and engineering of this shuttle in yeast and other eukaryotes.

## INTRODUCTION

In eukaryotes, metabolic compartmentation necessitates mechanisms for translocation of metabolites between cellular compartments. Acetyl coenzyme A (acetyl-CoA) is an important precursor in cytosolic and mitochondrial biosynthetic pathways and, moreover, is involved in cellular regulation by acting as an acetyl donor for acetylation of nuclear and cytosolic proteins (1–5). Eukaryotes have evolved several mechanisms for synthesis and intracellular transport of acetyl-CoA within and between cellular compartments (6–8). One of these mechanisms, the carnitine shuttle, plays a key role in translocation of acetyl units between cellular compartments during growth of *Saccharomyces cerevisiae* on fatty acids (9–11).

In contrast to the situation in mammals, in which fatty acid β-oxidation also occurs in mitochondria, this process is confined to peroxisomes in *S. cerevisiae* (12). Further metabolism of acetyl-CoA, the major product of fatty acid β-oxidation, requires transport of its acetyl moiety from peroxisomes to other cellular compartments (11). This transport is initiated by a peroxisomal carnitine acetyltransferase, which transfers the acetyl moiety of acetyl-CoA to l-carnitine, yielding acetyl-l-carnitine and coenzyme A. Acetyl-l-carnitine is then transported to other compartments, where carnitine acetyltransferases catalyze the reverse reaction, thereby regenerating acetyl-CoA and l-carnitine.

In *S. cerevisiae*, six proteins have been reported to contribute to the *in vivo* functionality of the carnitine shuttle. In contrast to many other eukaryotes, including mammals (13) and the yeast *Candida albicans* (14), *S. cerevisiae* lacks the genes required for l-carnitine biosynthesis (9, 15). As a consequence, operation of the carnitine shuttle in *S. cerevisiae* depends on import of exogenous l-carnitine via the Hnm1 plasma membrane transporter (16), whose expression is regulated by the plasma membrane protein Agp2 (16, 17). The three carnitine acetyltransferases in *S. cerevisiae* (11) have different subcellular localizations: Cat2 is active in the peroxisomal and mitochondrial matrices (18), Yat1 is localized to the outer mitochondrial membrane (19), and Yat2 has been reported to be cytosolic (15, 20, 21). The inner mitochondrial membrane contains an (acetyl-)carnitine translocase, Crc1 (17, 22–24), while export of acetyl-l-carnitine from peroxisomes has been proposed to occur via diffusion through channels in the peroxisomal membrane (25).

**FIG 1:**
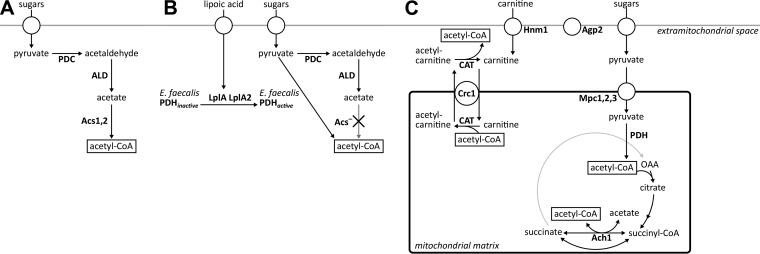
Cytosolic acetyl-CoA metabolism in (engineered) *Saccharomyces cerevisiae* strains. (A) In wild-type strains, cytosolic acetyl-CoA is produced via the PDH bypass, consisting of pyruvate carboxylase, acetaldehyde dehydrogenase, and acetyl-CoA synthetase. (B) Replacing the native route of acetyl-CoA synthesis by the *Enterococcus faecalis* PDH complex requires the extracellular addition of lipoic acid for activation of the E2 subunit of the cytosolically expressed bacterial PDH complex. (C) In the evolved strains IMS0482 and IMS0483, extracellular l-carnitine is imported into the mitochondria via the Hnm1 transporter at the plasma membrane and the Crc1 transporter at the inner mitochondrial membrane. Pyruvate is imported into the mitochondria, following its oxidative decarboxylation by the native mitochondrial PDH complex. The acetyl moiety is then transferred to l-carnitine, followed by export of acetyl-l-carnitine to the cytosol. There, carnitine acetyltransferases move the acetyl moiety back to CoA, yielding cytosolic acetyl-CoA. Abbreviations: Ach1, CoA transferase; Acs, Acs1, and Acs2, acetyl-CoA synthetase; Agp2, regulator; ALD, acetaldehyde dehydrogenase; CAT, carnitine acetyltransferase; Crc1, acetyl-carnitine translocase; Hnm1, carnitine transporter; LplA and LplA2, lipoylation proteins; Mpc1, Mpc2, and Mpc3, mitochondrial pyruvate carrier; OAA, oxaloacetate; PDC, pyruvate decarboxylase; PDH, pyruvate dehydrogenase complex.

Catabolism of the acetyl-CoA generated during growth of *S. cerevisiae* on fatty acids involves the mitochondrial tricarboxylic acid (TCA) cycle. Conversely, during growth on glucose, the mitochondria act as an important source of acetyl-CoA, with the pyruvate dehydrogenase (PDH) complex catalyzing the predominant acetyl-CoA generating reaction (8, 26). The carnitine acetyltransferase reaction is, in principle, mechanistically and thermodynamically reversible (Δ*G_R_*°′ = −1.1 kJ ⋅ mol^−1^ in the direction of acetyl-l-carnitine formation [27]). This observation suggests that the carnitine shuttle should not only be able to import acetyl units into the mitochondria but also be able to export them from the mitochondrial matrix to the cytosol. Therefore, based on *in vitro* experiments, it was initially hypothesized that the carnitine shuttle was responsible for export of acetyl moieties from yeast mitochondria (22). Further studies, however, indicated that the PDH bypass, which encompasses the concerted action of pyruvate decarboxylase, acetaldehyde dehydrogenase, and acetyl-CoA synthetase (28), was responsible for cytosolic acetyl-CoA provision in glucose-grown *S. cerevisiae* cultures (26) (Fig. 1A). Several additional observations argue against an *in vivo* role of the carnitine shuttle in export of acetyl moieties from mitochondria to cytosol in glucose-grown cultures. In wild-type *S. cerevisiae*, transcription of genes involved in the carnitine shuttle is strongly glucose repressed (18, 19, 29), which precludes a significant contribution to cytosolic acetyl-CoA provision in glucose-grown batch cultures. Moreover, even in derepressed, glucose-limited chemostat cultures, supplementation of growth media with l-carnitine cannot complement the growth defect of strains lacking a functional PDH bypass, which is caused by an inability to synthesize cytosolic acetyl-CoA (30). Hence, based on currently available data, the carnitine shuttle of *S. cerevisiae* appears to operate unidirectionally (i.e., transporting acetyl moieties into the mitochondria) during growth on glucose.

The goal of the present study is to investigate the molecular basis for the apparent unidirectionality of the yeast carnitine shuttle. To this end, we studied growth on glucose of an *S. cerevisiae* strain in which the carnitine shuttle is constitutively expressed. We recently demonstrated that constitutive expression of the components of the carnitine shuttle enables efficient transport of acetyl moieties from cytosol to mitochondria in glucose-grown, l-carnitine-supplemented batch cultures (8). In the present study, overexpression of the carnitine shuttle proteins was combined with replacement of the native *S. cerevisiae* pathway for cytosolic acetyl-CoA synthesis by a cytosolically expressed bacterial PDH complex (31). In the resulting strain, cytosolic acetyl-CoA synthesis could be switched off at will by omitting lipoic acid from growth media. After evolving in the laboratory, mutations required for l-carnitine-dependent growth in the absence of lipoic acid were identified by whole-genome sequencing and functionally analyzed by their introduction in the nonevolved parental strain.

## RESULTS

### Constitutive expression of carnitine shuttle genes does not rescue growth on glucose of *S. cerevisiae* *acs1*Δ *acs2*Δ strain.

**FIG 2:**
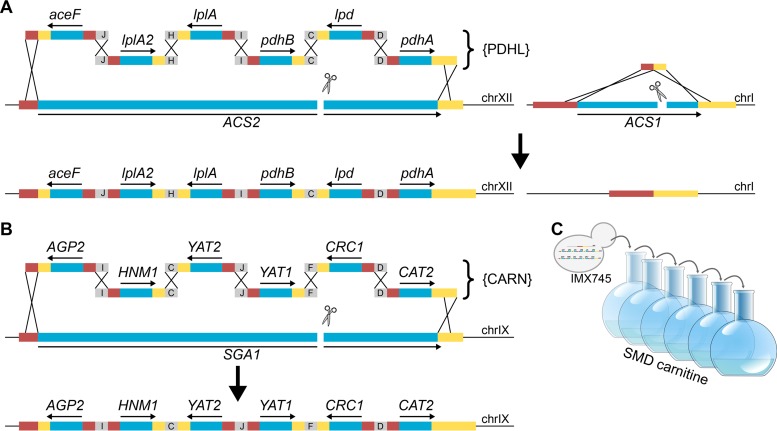
Construction of a lipoic acid-dependent, carnitine shuttle-constitutive *S. cerevisiae* strain and its laboratory evolution for lipoic acid-independent, carnitine-dependent growth. (A) In a previous study (33), the {PDHL} cluster, consisting of six cassettes required for cytosolic expression of a functional *Enterococcus faecalis* pyruvate dehydrogenase complex and flanked by 60-bp sequences, was assembled *in vivo* via homologous recombination (indicated with black crosses) and introduced in *ACS2* after introduction of a Cas9-induced double-strand break. *ACS1* was removed using a 120-bp DNA repair fragment (figure adapted from reference 33). (B) In this strain, the {CARN} cluster, consisting of six cassettes for constitutive expression of carnitine shuttle genes, was similarly *in vivo* assembled and introduced into the *SGA1* locus, resulting in strain IMX745 (*acs1*Δ *acs2*Δ::{PDHL} *sga1*Δ::{CARN}). Activity of the *E. faecalis* PDH in the yeast cytosol is lipoic acid dependent (31). (C) As strain IMX745 did not show l-carnitine-dependent growth when lipoic acid was omitted from growth media, an evolution experiment was initiated using synthetic medium with 20 g ⋅ liter^−1^ glucose (dextrose) (SMD) and 400 mg ⋅ liter^−1^ l-carnitine. Abbreviations: chrI, chromosome I; chrIX, chromosome IX; chrXII, chromosome XII.

Interpretation of previous studies on the role of the carnitine shuttle in glucose-grown cultures of *S. cerevisiae* is complicated by the strong glucose repression of the structural genes encoding carnitine acetyltransferases and acetyl-carnitine translocase (18, 19, 29, 32). To reexamine whether the carnitine shuttle can translocate acetyl units from mitochondria to cytosol, a strain was constructed in which provision of cytosolic acetyl-CoA could be made strictly dependent on a constitutively expressed carnitine shuttle. Its construction (Fig. 2A) started with a strain in which cytosolic acetyl-CoA metabolism had been modified by replacing the acetyl-CoA synthetase genes *ACS1* and *ACS2* by the six-gene {PDHL} cluster (we use the curly brackets to indicate a chromosomally integrated cluster of PDH complex {PDHL} genes as discussed in “Strain construction” below in Materials and Methods) (33) (Table 1), which enables functional expression in the yeast cytosol of the *Enterococcus faecalis* PDH complex (Fig. 1B). This strain provided an experimental model in which cytosolic acetyl-CoA synthesis could be switched off at will by omitting lipoic acid from growth media. The functionality of alternative (introduced) routes to cytosolic acetyl-CoA could thus be tested by omitting lipoic acid and checking for growth. Expression cassettes were constructed in which the yeast carnitine shuttle genes (*AGP2*, *CAT2*, *CRC1*, *HNM1*, *YAT1*, and *YAT2*) were controlled by strong, constitutive promoters. The resulting six DNA fragments were assembled and integrated as a single cluster of carnitine genes ({CARN}; Fig. 2B; Table 1) into the genome of the strain carrying the {PDHL} cluster. Consistent with an earlier study on cytosolic expression of the *E. faecalis* PDH complex in *S. cerevisiae* (31), growth of the resulting strain IMX745 (*acs1*Δ *acs2*Δ::{PDHL} *sga1*Δ::{CARN}) on synthetic medium containing glucose depended on the addition of lipoic acid to the growth medium.

**TABLE 1:** *Saccharomyces cerevisiae* strains used in this study

Strain	Relevant genotype^a^	Parental strain(s)	Source or reference
CEN.PK113-7D	*MAT***a**		P. Kötter
IMX585	*MAT***a** *can1*Δ::*cas9-natNT2*	CEN.PK113-7D	33
IMX719	*MAT***a** *can1*Δ::*cas9-natNT2 acs1Δ acs2*Δ::{PDHL}	IMX585	33
IMX868	*MAT*α *can1*Δ::*cas9-natNT2 sga1*Δ::{CARN}		8
IMX745	*MAT***a** *can1*Δ::*cas9-natNT2 acs1Δ acs2*Δ::{PDHL} *sga1*Δ::{CARN}	IMX719	This study
IMS0482	*MAT***a** *can1*Δ::*cas9-natNT2 acs1Δ acs2*Δ::{PDHL} *sga1*Δ::{CARN}	IMX745	This study
IMS0483	*MAT***a** *can1*Δ::*cas9-natNT2 acs1Δ acs2*Δ::{PDHL} *sga1*Δ::{CARN}	IMX745	This study
IMW074	*MAT***a** *can1*Δ::*cas9-natNT2 acs1Δ acs2*Δ::{PDHL} *sga1*Δ	IMS0482	This study
IMW075	*MAT***a** *can1*Δ::*cas9-natNT2 acs1Δ acs2Δ sga1*Δ::{CARN}	IMS0482	This study
IMW076	*MAT***a** *can1*Δ::*cas9-natNT2 acs1Δ acs2*Δ::{PDHL} *sga1*Δ	IMS0483	This study
IMW077	*MAT***a** *can1*Δ::*cas9-natNT2 acs1Δ acs2Δ sga1*Δ::{CARN}	IMS0483	This study
IMW078	*MAT***a** *can1*Δ::*cas9-natNT2 acs1Δ acs2*Δ::{PDHL} *sga1*Δ::{CARN} *ach1*Δ	IMS0482	This study
IMW079	*MAT***a** *can1*Δ::*cas9-natNT2 acs1Δ acs2*Δ::{PDHL} *sga1*Δ::{CARN} *pda1*Δ	IMS0482	This study
IMW081	*MAT***a** *can1*Δ::*cas9-natNT2 acs1Δ acs2*Δ::{PDHL} *sga1*Δ::{CARN} *ach1*Δ	IMS0483	This study
IMW082	*MAT***a** *can1*Δ::*cas9-natNT2 acs1Δ acs2*Δ::{PDHL} *sga1*Δ::{CARN} *pda1*Δ	IMS0483	This study
IMX847	*MAT***a** *can1*Δ::*cas9-natNT2 acs1Δ acs2*Δ::{PDHL} *sga1*Δ::{CARN} *MCT1^T641G^*	IMX745	This study
IMX849	*MAT***a** *can1*Δ::*cas9-natNT2 acs1Δ acs2*Δ::{PDHL} *sga1*Δ::{CARN} *RTG2^G503T^*	IMX745	This study
IMX852	*MAT***a** *can1*Δ::*cas9-natNT2 acs1Δ acs2*Δ::{PDHL} *sga1*Δ::{CARN} *MCT1^T641G^ RTG2^G503T^*	IMX745	This study
IMX907	*MAT***a** *can1*Δ::*cas9-natNT2 acs1Δ acs2*Δ::{PDHL} *sga1*Δ::{CARN, *pADH1-YAT2^C173G^*}	IMX745	This study
IMX909	*MAT***a** *can1*Δ::*cas9-natNT2 acs1Δ acs2*Δ::{PDHL} *sga1*Δ::{CARN,*pADH1-YAT2^C173G^*} *MCT1^T641G^*	IMX847	This study
IMX911	*MAT***a** *can1*Δ::*cas9-natNT2 acs1Δ acs2*Δ::{PDHL} *sga1*Δ::{CARN,*pADH1-YAT2^C173G^*} *RTG2^G503T^*	IMX849	This study
IMX913	*MAT***a** *can1*Δ::*cas9-natNT2 acs1Δ acs2*Δ::{PDHL} *sga1*Δ::{CARN,*pADH1-YAT2^C173G^*} *MCT1^T641G^ RTG2^G503T^*	IMX852	This study
IMX932	*MAT***a** *can1*Δ::*cas9-natNT2 acs1Δ acs2*Δ::{PDHL} *sga1*Δ::{CARN,*yat2*Δ} *MCT1^T641G^ RTG2^G503T^*	IMX852	This study
IMX933	*MAT***a** *can1*Δ::*cas9-natNT2 acs1Δ acs2*Δ::{PDHL} *sga1*Δ::{CARN,*pADH1-YAT2^C173G^*} *MCT1^T641G^* *rtg2*Δ	IMX909	This study
IMX934	*MAT***a** *can1*Δ::*cas9-natNT2 acs1Δ acs2*Δ::{PDHL} *sga1*Δ::{CARN,*pADH1-YAT2^C173G^*} *mct1Δ RTG2^G503T^*	IMX911	This study
IMX923	*MAT***a** *can1*Δ::*cas9-natNT2 sga1*Δ::*pADH1-YAT2-tYAT2*	IMX585	This study
IMX925	*MAT***a** *can1*Δ::*cas9-natNT2 sga1*Δ::*pADH1-YAT2^C173G^-YAT2*	IMX585	This study
CEN.PK122	*MAT***a**/*MAT*α		P. Kötter
CEN.PK194-2C	*MAT***a** *cat2*Δ::*loxP-KanMX4-loxP*	CEN.PK122	This study
CEN.PK196-2C	*MAT*α *yat1*Δ::*loxP-KanMX4-loxP*	CEN.PK122	This study
CEN.PK215-4A	*MAT***a** *cat2*Δ::*loxP-KanMX4-loxP yat1*Δ::*loxP-KanMX4-loxP*	CEN.PK194-2C × CEN.PK196-2C	This study
CEN.PK113-5D	*MAT***a** *ura3-52*		P. Kötter
IME140	*MAT***a** *ura3-52* p426GPD (2µm ori *URA3*)	CEN.PK113-5D	58
IME320	*MAT***a** *ura3-52* pUDE390 (2µm ori *URA3 pADH1-YAT2-tYAT2*)	CEN.PK113-5D	This study
IME321	*MAT***a** *ura3-52* pUDE391 (2µm ori *URA3 pADH1-YAT2^C173G^*-tYAT2)	CEN.PK113-5D	This study
IME233	*MAT***a** *ura3-52* pUDE336 (2µm ori *URA3 pTDH3-CAT2-His_6_-tCYC1*)	CEN.PK113-5D	This study

a The *RTG2^G503T^* mutation translates into an Rtg2^W168L^ protein, the *MCT1^T641G^* mutation translates into an Mct1^L214W^ protein, and the *YAT2^C173G^* mutation translates into an Yat2^P58R^ protein. {PDHL}, chromosomally integrated *E. faecalis* PDH gene cluster, *pADH1-aceF-tPGI1 pPGI1-lplA2-tPYK1 pPGK1-lplA-tPMA1 pTDH3-pdhB-tCYC1 pTEF1-lpd-tADH1 pTPI1-pdhA-tTEF1*. {CARN}, *pTDH3-AGP2-tAGP2 pPGK1-HNM1-tHNM1 pADH1-YAT2-tYAT2 pPGI1-YAT1-tYAT1 pTPI1-CRC1-tCRC1 pTEF1-CAT2-tCAT2*.

**FIG 3:**
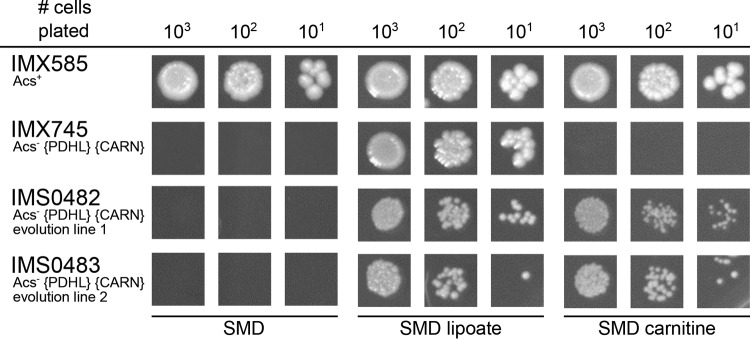
Growth on glucose of *S. cerevisiae* strains in the presence and absence of lipoic acid and l-carnitine. *S. cerevisiae* strains were pregrown in shake flasks on synthetic medium with 20 g ⋅ liter^−1^ glucose (strain IMX585), supplemented with lipoic acid (strain IMX745) or l-carnitine (strains IMS0482 and IMS0483) and spotted on plates containing synthetic medium with glucose (dextrose) without lipoic acid or l-carnitine (SMD), with lipoic acid (SMD lipoate), and with l-carnitine (SMD carnitine). The plates were incubated for 100 h at 30°C. Relevant strain descriptions are given in the figure. Photographs of the entire spot plates are shown in Data Set S1 in the supplemental material.

Enzyme activities in cell extracts of strain IMX745 showed a carnitine acetyltransferase (CAT) activity of 3.2 ± 0.1 µmol ⋅ mg protein^−1^ ⋅ min^−1^, while activities in extracts of the parental strain IMX719 (*acs1*Δ *acs2*Δ::{PDHL}) and of the reference strain IMX585 (*ACS1 ACS2*) were below the detection limit of the assay (<0.01 µmol ⋅ mg protein^−1^ ⋅ min^−1^). Growth of strain IMX745 was not observed when lipoic acid was replaced by l-carnitine or when both growth factors were omitted from the glucose-containing synthetic medium (Fig. 3). This result demonstrated that, even when constitutively expressed, the *S. cerevisiae* carnitine shuttle cannot export acetyl units from mitochondria at a rate that is sufficient to meet cytosolic acetyl-CoA requirements in an *acs1*Δ *acs2*Δ strain background.

### Laboratory evolution yields mutants in which the carnitine shuttle provides cytosolic acetyl-CoA.

**FIG 4:**
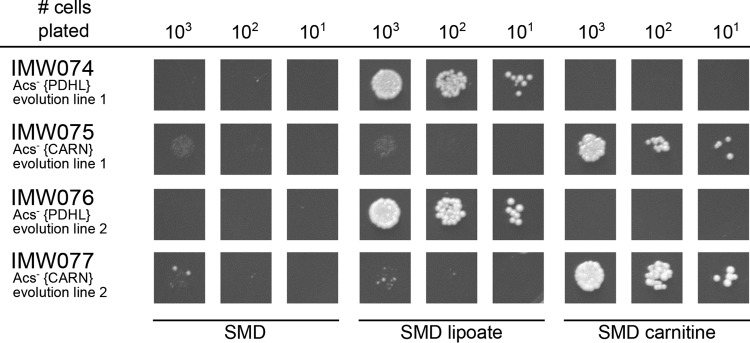
Growth on glucose of *S. cerevisiae* strains in the presence and absence of lipoic acid and l-carnitine. *S. cerevisiae* strains were pregrown in shake flasks on synthetic medium with 20 g ⋅ liter^−1^ glucose, supplemented with lipoic acid (strains IMW074 and IMW076) or l-carnitine (strains IMW075 and IMW077) and spotted on plates containing synthetic medium with glucose (dextrose) without lipoic acid or l-carnitine (SMD), with lipoic acid (SMD lipoate) and with l-carnitine (SMD carnitine). The plates were incubated for 100 h at 30°C. Relevant strain descriptions are given in the figure. Photographs of the entire spot plates are shown in Data Set S1 in the supplemental material.

To investigate whether laboratory evolution can enable the carnitine shuttle to support export of acetyl units from the mitochondrial matrix, a laboratory evolution experiment was started with strain IMX745 (Acs^−^ {PDHL} {CARN}) by starting two independent shake flask cultures on synthetic medium with 20 g ⋅ liter^−1^ glucose and 400 mg ⋅ liter^−1^ l-carnitine (Fig. 2C). Following 2 weeks of incubation, growth was observed in both shake flasks, and after six or seven subsequent transfers (corresponding to ca. 70 generations), single-cell lines were isolated from each experiment, resulting in strains IMS0482 and IMS0483. These two evolved strains readily grew on glucose-containing synthetic medium supplemented with either lipoic acid or l-carnitine, but they did not grow when both compounds were omitted from the medium (Fig. 3). In shake flask cultures on glucose-containing synthetic medium, addition of l-carnitine supported specific growth rates of 0.14 h^−1^ (IMS0482) and 0.10 h^−1^ (IMS0483) (Table 2). When the synthetic gene cluster encoding the *E. faecalis* PDH complex {PDHL} was removed from the evolved strains, growth of the resulting strains on glucose could no longer be supported by the addition of lipoic acid and, instead, became uniquely dependent on l-carnitine (Fig. 4). Conversely, deletion of the six carnitine shuttle expression cassettes {CARN} from the evolved strains abolished their l-carnitine-dependent growth, leaving the strains uniquely dependent on lipoic acid (Fig. 4). Together, these results unequivocally show that, in the evolved strains, export of the acetyl moiety of mitochondrially produced acetyl-CoA via the constitutively expressed carnitine shuttle supported cytosolic acetyl-CoA provision (Fig. 1C).

**TABLE 2:** Specific growth rates of different *S. cerevisiae* *acs1*Δ *acs2*Δ strains on glucose in the presence of l-carnitine^a^

Strain	Short description^b^	Growth rate (h^−1^)^c^
IMX745	Unevolved strain	No growth^d^
IMS0482	Evolution line 1	0.14
IMS0483	Evolution line 2	0.10
IMX909	Mct1^L214W^ Rtg2 Yat2^P58R^	0.10−0.06^e^
IMX913	Mct1^L214W^ Rtg2^W168L^ Yat2^P58R^	0.14

a *S. cerevisiae* Acs^−^ strains were grown on synthetic medium containing glucose but lacking lipoic acid, thereby blocking synthesis of cytosolic acetyl-CoA via heterologously expressed bacterial pyruvate dehydrogenase complex. Strains were grown in shake flasks with 20 g ⋅ liter^−1^ glucose; media were supplemented with 40 mg ⋅ liter^−1^ l-carnitine.

b All strains harbor the {PDHL} and {CARN} gene sets. Composition of these gene sets is described in Materials and Methods.

c The growth rates shown are averages of two independent experiments for each strain. With the exception of strain IMX909, which showed biphasic growth, the average deviation of the mean specific growth rate was ≤0.01 h^−1^ in all experiments.

d Growth was observed only in the presence of lipoic acid (0.29 h^−1^).

e Shake flask cultures of strain IMX909 showed decelerating growth rates from mid-exponential phase onward.

### The mitochondrial PDH complex is the predominant source of acetyl-CoA in evolved, l-carnitine-dependent *acs1*Δ *acs2*Δ strains.

**FIG 5:**
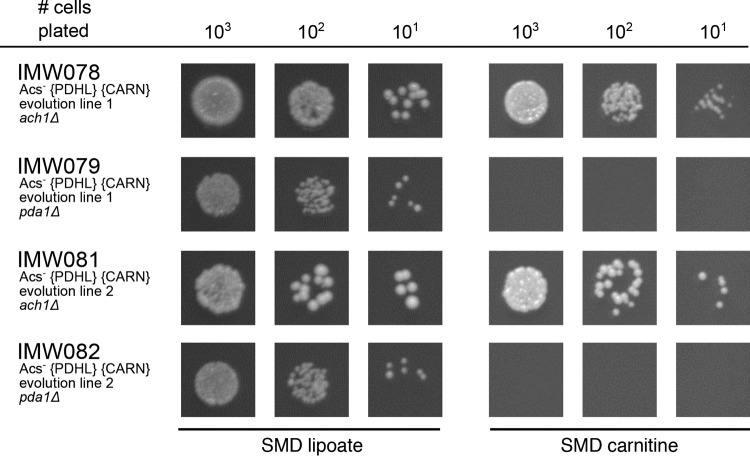
Growth on glucose of *S. cerevisiae* strains in the presence of lipoic acid or l-carnitine. *S. cerevisiae* strains were pregrown in shake flasks on synthetic medium with 20 g ⋅ liter^−1^ glucose, supplemented with lipoic acid and spotted on plates containing synthetic medium with glucose (dextrose) and with lipoic acid (SMD lipoate) or with l-carnitine (SMD carnitine). The plates were incubated for 100 h at 30°C. Relevant strain descriptions are given in the figure. Photographs of the entire spot plates are shown in Data Set S1 in the supplemental material.

In *S. cerevisiae*, mitochondrial acetyl-CoA can be generated by the native, mitochondrial PDH complex and by the mitochondrial succinyl-CoA:acetate CoA-transferase Ach1 (8, 26, 34). To study which of these reactions provided mitochondrial acetyl-CoA in the evolved strains IMS0482 and IMS0483, the mitochondrial PDH complex was inactivated by deleting *PDA1* (35, 36), and Ach1 activity was abolished by disrupting *ACH1*. In both evolved strains, deletion of *PDA1* abolished l-carnitine-dependent growth on glucose, while *ACH1* disruption did not have a detectable impact on growth (Fig. 5). These results demonstrate that, in glucose-grown batch cultures of the evolved strains, the *S. cerevisiae* PDH complex is the predominant source of mitochondrial acetyl-CoA and, via the constitutively expressed carnitine shuttle, of cytosolic acetyl-CoA.

### Whole-genome sequencing and reverse engineering of evolved l-carnitine-dependent strains.

To identify the mutations that enabled l-carnitine-dependent growth of the evolved carnitine-dependent *acs1*Δ *acs2*Δ strains, the genomes of strains IMS0482 and IMS0483 (Acs^−^ {PDHL} {CARN}, isolated from evolution lines 1 and 2, respectively) and of their parental strain IMX745 (Acs^−^ {PDHL} {CARN}) were sequenced. Analysis of single-nucleotide changes and insertions/deletions (indels) in open reading frames revealed only three mutations in strain IMS0482 (evolution line 1) and four mutations in strain IMS0483 (evolution line 2) relative to the parental strain (Table 3). Analysis of copy number variations (37) showed that strain IMS0482 carried a duplication of chromosome X (data not shown). Chromosome X did not carry either one of the two synthetic gene clusters or any of three mutated genes. No copy number variations relative to the parental strain were detected in strain IMS0483.

**TABLE 3:** Mutations in evolved *S. cerevisiae* strains with l-carnitine-dependent provision of cytosolic acetyl-CoA^a^**

Strain and gene	Nucleotide change	Amino acid change	Description
IMS0482			
*RTG2*	G503T	W168L	Sensor of mitochondrial dysfunction; regulates the subcellular location of Rtg1p and Rtg3p, transcriptional activators of the retrograde (RTG) and target of rapamycin (TOR) pathways; Rtg2p is inhibited by the phosphorylated form of Mks1p
*MCT1*	T641G	L214W	Predicted malonyl-CoA:ACP transferase; putative component of a type II mitochondrial fatty acid synthase that produces intermediates for phospholipid remodeling
*YAT2*	C173G	P58R	Carnitine acetyltransferase; has similarity to Yat1p, which is a carnitine acetyltransferase associated with the mitochondrial outer membrane
IMS0483			
*RPO21*	A2507G	Y836C	RNA polymerase II largest subunit B220; part of central core; phosphorylation of C-terminal heptapeptide repeat domain regulates association with transcription and splicing factors; similar to bacterial beta-prime
*HXT6* or *HXT7*	Gene deletion	Gene deletion	High-affinity glucose transporter; member of the major facilitator superfamily, nearly identical to Hxt7p, expressed at high basal levels relative to other HXTs, repression of expression by high glucose requires *SNF3*
*STB2*	C1073A	P358Q	Protein that interacts with Sin3p in a two-hybrid assay; part of a large protein complex with Sin3p and Stb1p; *STB2* has a paralog, *STB6*, that arose from the whole-genome duplication
*MCT1*	C292T	Q98*	Predicted malonyl-CoA:ACP transferase; putative component of a type II mitochondrial fatty acid synthase that produces intermediates for phospholipid remodeling

a Mutations in the open reading frames of the laboratory-evolved strains IMS0482 and IMS0483 were identified by comparing whole-genome sequence data to those of the unevolved parental strain IMX745. Descriptions of gene function were obtained from the *Saccharomyces* Genome Database website (76).

Both evolved strains carried mutations in *MCT1*, which is predicted to encode the mitochondrial malonyl-CoA:acyl carrier protein (ACP) transferase that catalyzes the second step of mitochondrial fatty acid synthesis (21, 38, 39). In strain IMS0482, the T-to-G change at position 641 encoded by *MCT1* (*MCT1^T641G^*) caused an amino acid change from leucine to tryptophan at position 214, and in strain IMS0483, an *MCT1^C292T^* mutation caused a premature stop codon at position 98. Strain IMS0482 carried an additional mutation in *RTG2*, which resulted in a W168L amino acid change. Rtg2 is involved in communication between mitochondria and the nucleus, and deletion of *RTG2* negatively affects activity of citrate synthase (oxaloacetate + acetyl-CoA + H_2_O → citrate + CoA; 40, 41). A third mutation in strain IMS0482 was found in the introduced expression cassette for *YAT2*, which has been reported to encode a cytosolic carnitine acetyltransferase (15) and caused a P58R amino acid change in the evolved strain. In strain IMS0483, the abovementioned *MCT1^C292T^* mutation was accompanied by single-nucleotide changes in the coding regions of *RPO21* and *STB2* and a deletion of either *HXT6* or *HXT7*. Since the protein products of these three genes did not show an obvious relation with mitochondrial metabolism (Table 3), further analysis was focused on the mutations found in strain IMS0482 which, moreover, exhibited the highest specific growth rate on glucose of the two evolved strains (Table 2).

### Mutations in *MCT1*, *RTG1*, and *YAT2* together enable *in vivo* reversal of the mitochondrial carnitine shuttle.

**FIG 6:**
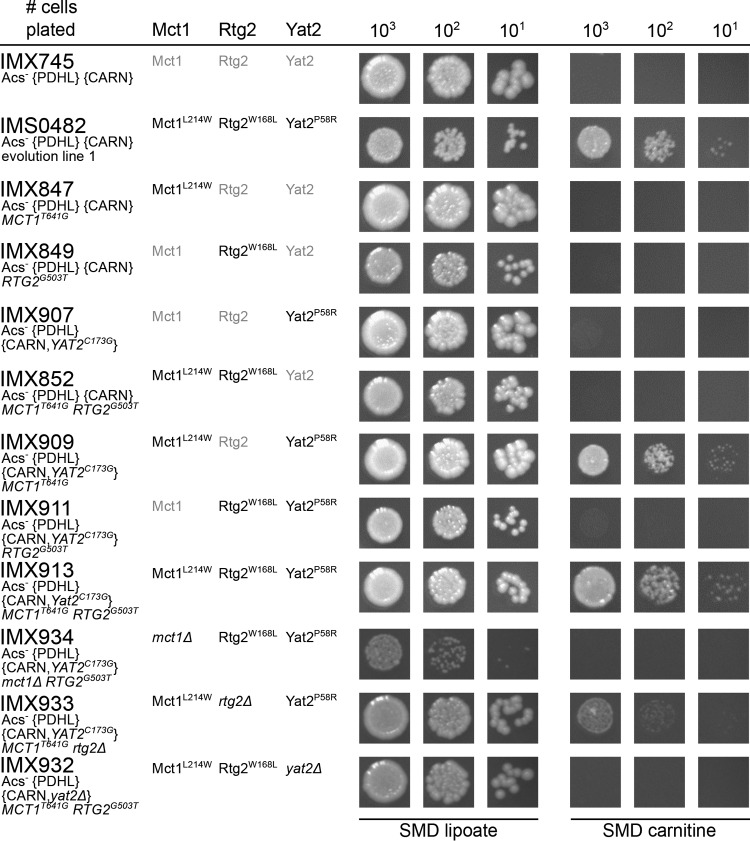
Growth on glucose of *S. cerevisiae* strains in the presence of lipoic acid or l-carnitine. *S. cerevisiae* strains were pregrown in shake flasks on synthetic medium with 20 g ⋅ liter^−1^ glucose, supplemented with lipoic acid and spotted on plates containing synthetic medium with glucose (dextrose) and with lipoic acid (SMD lipoate) or with l-carnitine (SMD carnitine). The plates were incubated for 100 h at 30°C. Relevant strain descriptions are given in the figure. Photographs of the entire spot plates are shown in Data Set S1 in the supplemental material.

**FIG 7:**
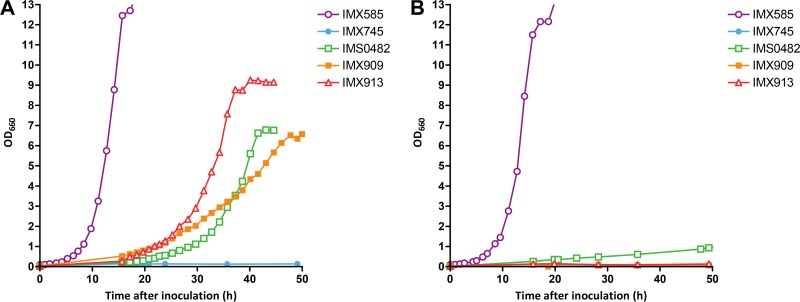
Growth curves of *S. cerevisiae* strains. *S. cerevisiae* strains IMX585 (Acs^+^ reference), IMX745 (Acs^−^ {PDHL} {CARN}), IMS0482 (Acs^−^ {PDHL} {CARN}, evolution line 1), IMX909 (Acs^−^ {PDHL} {CARN,*pADH1-YAT2^C173G^*} *MCT1^T641G^*), and IMX913 (Acs^−^ {PDHL} {CARN,*pADH1-YAT2^C173G^*} *MCT1^T641G^ RTG2^G503T^*) were grown on synthetic medium containing glucose with or without l-carnitine. All strains were pregrown in liquid synthetic medium with 20 g⋅ liter^−1^ glucose and lipoic acid, washed with synthetic medium, and transferred to new shake flasks with synthetic medium containing 20 g⋅ liter^−1^ glucose. (A) Cultures supplemented with l-carnitine, (B) cultures without l-carnitine. Values are averages and mean deviations (error bars were smaller than size of symbols) from single shake flask experiments that are quantitatively representative of duplicate experiments.

To investigate their biological relevance, the three mutations found in evolved strain IMS0482 were introduced individually and in different combinations into the nonevolved parental strain IMX745 (Acs^−^ {PDHL} {CARN}). As expected, all resulting strains grew on synthetic medium with glucose and lipoic acid. However, on solid medium, only strains IMX909 (Mct1^L214W^ Rtg2 Yat2^P58R^) and IMX913 (Mct1^L214W^ Rtg2^W168L^ Yat2^P58R^) showed l-carnitine-dependent growth (Fig. 6), suggesting that both Mct1^L214W^ and Yat2^P58R^ were essential for the acquired phenotype. On spot plates, no clear impact of the mutation in *RTG2* was observed after 100 h of incubation (Fig. 6). For a quantitative analysis of the impact of the Rtg2^W168L^ mutation on specific growth rates, strains IMX909 (Mct1^L214W^ Rtg2 Yat2^P58R^) and IMX913 (Mct1^L214W^ Rtg2^W168L^ Yat2^P58R^) were grown in shake flask cultures on synthetic medium with glucose and l-carnitine (Table 2 and Fig. 7). Strain IMX909 showed decelerating exponential growth rates of 0.10 h^−1^ to 0.06 h^−1^, while strain IMX913 exhibited monophasic exponential growth at a specific growth rate of 0.14 h^−1^, which resembled the specific growth rate of evolved strain IMS0482 (Fig. 7). This result showed that all three mutations in the laboratory-evolved strain IMS0482 contributed to its acquired phenotype. Exponentially growing cultures of the reverse engineered strain IMX913 on synthetic medium with glucose and l-carnitine exhibited a high viability (>99%), resembling that of the reference strain IMX585.

To investigate whether the mutations in *MCT1*, *RTG2*, and *YAT2*, acquired by strain IMS0482 during laboratory evolution, might have caused a complete loss of function, three Acs^−^ {PDHL} {CARN} strains were constructed in which deletion of one of the three genes was combined with the acquired point mutations of the remaining two genes. The three resulting strains, IMX932, IMX933, and IMX934, all showed growth after 100-h incubation on solid medium with glucose and lipoic acid (Fig. 6). However, strains IMX934 (Acs^−^ {PDHL} {CARN,Yat2^P58R^} *mct1*Δ Rtg2^W168L^) and IMX932 (Acs^−^ {PDHL} {CARN,*yat2*Δ} Mct1^L214W^ Rtg2^W168L^) were unable to grow on medium with l-carnitine, while strain IMX933 (Acs^−^ {PDHL} {CARN,Yat2^P58R^} Mct1^L214W^ *rtg2*Δ) did show l-carnitine-dependent growth (Fig. 6). This result indicated that the amino acid changes in the Mct1^L214W^ and Yat2^P58R^ variants did not result in complete loss of function. Interestingly, the genetic context of the other evolved strain IMS0483, in which *MCT1* contained a premature stop codon, did appear to enable carnitine-dependent growth in the absence of a functional Mct1 protein. The slightly lower l-carnitine-dependent growth of strain IMX933 (Acs^−^ {PDHL} {CARN,Yat2^P58R^} Mct1^L214W^ *rtg2*Δ) compared to a congenic strain expressing the mutant Rtg2^W168L^ variant, suggests that this amino acid change does not lead to a completely nonfunctional protein.

### Enzyme assays do not confirm carnitine acetyltransferase activity of Yat2.

The prior classification of Yat2 as a cytosolic carnitine acetyltransferase (20, 21, 24) was based on its homology with other carnitine acetyltransferase genes and on a reported 50% decrease of carnitine acetyltransferase activity (not normalized for protein content) in cell extracts of ethanol-grown cultures of a *yat2*Δ strain (15). To compare carnitine acetyltransferase activities of Yat2 and Yat2^P58R^, *YAT2* and *YAT2^C173G^* genes under control of the constitutive *ADH1* promoter were introduced in reference genetic backgrounds. Since the native *YAT1*, *YAT2*, and *CAT2* carnitine acetyltransferases are repressed by glucose, enzyme assays on cell extracts of glucose-grown batch cultures should reflect activity of only these constitutively expressed *YAT2* genes. Surprisingly, no detectable (<0.01 µmol ⋅ mg protein^−1^ ⋅ min^−1^) carnitine acetyltransferase activity was found in such experiments with strains expressing the wild-type *YAT2* or evolved alleles of *YAT2* from single-copy or multicopy, *pADH1*-controlled expression cassettes (Table 4). The same negative results were obtained with the carnitine acetyltransferase assay procedure described by Swiegers et al. (15). In contrast, strains IMX868 (*sga1*Δ::{CARN}) and IME233 (multicopy plasmid with constitutively expressed *CAT2*) showed high activities (Table 4). To exclude the theoretical possibility that Yat2 is subject to glucose catabolite inactivation, a *yat1*Δ *cat2*Δ *YAT2* strain (CEN.PK215-4A) was constructed and subsequently tested under glucose-derepressed, respiratory growth conditions. However, in ethanol-grown cultures of this strain, the Yat2-dependent carnitine acetyltransferase activity remained below the detection limit. Under the same conditions, the reference strain CEN.PK113-7D showed a carnitine acetyltransferase activity of 1.75 µmol ⋅ mg protein^−1^ ⋅ min^−1^ (Table 4).

**TABLE 4:** Specific carnitine acetyltransferase activities in cell extracts of *S. cerevisiae* strains^a^

Strain	Short description^b^	Carbon source in the medium	Carnitine acetyltransferase activity (µmol ⋅ mg protein^−1^ ⋅ min^−1^)**^c^
IMX585	Reference strain	Glucose	BD
IMX868	{CARN}	Glucose	2.69 ± 0.51
IMX923	*sga1*Δ::*pADH1-YAT2*	Glucose	BD
IMX925	*sga1*Δ::*pADH1-YAT2^C173G^*	Glucose	BD
IME140	Empty multicopy plasmid	Glucose	BD
IME320	Multicopy plasmid *pADH1-YAT2*	Glucose	BD
IME321	Multicopy plasmid *pADH1-YAT2^C173G^*	Glucose	BD
IME233	Multicopy plasmid *pTDH3-CAT2*	Glucose	4.24 ± 0.52
CEN.PK113-7D	*CAT2 YAT1 YAT2*	Ethanol	1.75 ± 0.02
CEN.PK215-4A	*cat2*Δ *yat1*Δ *YAT2*	Ethanol	BD
IMX745	{CARN}	Glucose	3.19 ± 0.14
IMS0482	{CARN} evolution line 1	Glucose	2.39 ± 0.05
IMX852	{CARN,*pADH1-YAT2*} *MCT1^T641G^RTG2^G503T^*	Glucose	2.92 ± 0.73
IMX913	{CARN,*pADH1-YAT2^C173G^*} *MCT1^T641G^ RTG2^G503T^*	Glucose	3.11 ± 0.71
IMX932	{CARN,*yat2*Δ} *MCT1^T641G^ RTG2^G503T^*	Glucose	2.82 ± 0.44

a Strains were grown in shake flasks containing synthetic medium with either 20 g ⋅ liter^−1^ glucose or 2% (vol/vol) ethanol as the carbon source and harvested in mid-exponential phase.

b The composition of the {CARN} gene set is described in Materials and Methods.

c Carnitine acetyltransferase activities in cell extracts were obtained from duplicate growth experiments and are shown as means ± standard deviations. The detection limit of the enzyme assay was 0.01 µmol ⋅  mg protein^−1^ ⋅ min^−1^. BD, below detection.

Possible explanations for our inability to detect Yat2-dependent carnitine acetyltransferase activity include the following. (i) Yat2 is active within a heteromeric complex only when another carnitine acetyltransferase is present. (ii) Yat2 is a catalytically inactive regulator of other carnitine acetyltransferases. (iii) Assay conditions and/or Yat2 protein instability preclude accurate measurement of *in vitro* Yat2 carnitine acetyltransferase activity. In the first two scenarios, the mutated form of Yat2 might still show a detectable impact on total carnitine acetyltransferase activity. However, while enzyme assays on cell extracts of strains IMX745 ({PDHL} {CARN}), IMS0482 ({PDHL} {CARN} evolution line 1), IMX852 ({PDHL} {CARN, Yat2} Mct1^L214W^ Rtg2^W168L^), IMX913 ({PDHL} {CARN, Yat2^P58R^} Mct1^L214W^ Rtg2^W168L^), and IMX932 ({PDHL} {CARN, *yat2*Δ} Mct1^L214W^ Rtg2^W168L^) all showed substantial carnitine acetyltransferase activities, the various strains did not show marked differences (Table 4).

## DISCUSSION

### Requirements for reversal of the mitochondrial carnitine shuttle.

To our knowledge, this study is the first to demonstrate that the carnitine shuttle can connect the mitochondrial acetyl-CoA pool to cytosolic, acetyl-CoA-consuming pathways in a eukaryote. Three requirements had to be met to enable export of acetyl units from mitochondria of glucose-grown *S. cerevisiae*. l-Carnitine, which cannot be synthesized by *S. cerevisiae* (9, 15), needed to be added to growth media. Furthermore, glucose repression of key genes encoding carnitine shuttle proteins had to be circumvented, which in this study was done by expression from constitutive promoters. While these first two criteria also have to be met to enable the carnitine shuttle to effectively import acetyl units into mitochondria (8, 9, 11, 15), its operation in the reverse direction additionally required mutations in the yeast genome.

Single-amino-acid changes in three proteins (Mct1^L214W^, Rtg2^W168L^, and Yat2^P58R^) together enabled export of acetyl units from mitochondria via a constitutively expressed carnitine shuttle. Mct1 is predicted to encode mitochondrial malonyl-CoA:ACP transferase (38), which is required for mitochondrial fatty acid synthesis. This process uses mitochondrial acetyl-CoA as a precursor and might therefore compete for this substrate with the carnitine shuttle. Mct1 uses malonyl-CoA, formed by the mitochondrial acetyl-CoA carboxylase Hfa1 (42), rather than acetyl-CoA, as a substrate. Inhibition of Hfa1 by malonyl-CoA, a property shared by several acetyl-CoA carboxylases (43, 44), could decrease its ability to compete for acetyl-CoA when Mct1 functions suboptimally. Rtg2, a sensor protein involved in the retrograde regulation pathway for nuclear-mitochondrial communication (40), was previously shown to affect levels of mitochondrial citrate synthase (41), which also uses mitochondrial acetyl-CoA as a substrate. We therefore propose that, in the evolved strains, mutations in *MCT1* and *RTG2* improved the driving force and/or kinetics of the export of acetyl units via the mitochondrial carnitine shuttle by negatively affecting pathways that compete for its substrate, intramitochondrial acetyl-CoA.

Mutations in mitochondrial lipid synthesis were previously shown to affect carnitine shuttle activity in human cells. When mitochondrial β-oxidation of fatty acids in human cells is compromised, acyl-carnitines are exported from the mitochondria to the cytosol and can even be found in blood plasma (45, 46). Especially when yeast carnitine shuttle genes can be functionally replaced by their human orthologs (47), the l-carnitine-dependent strains described in this study provide interesting platforms for studying the role of the carnitine shuttle in healthy and diseased human cells.

Many eukaryotes use a citrate-oxaloacetate shuttle, consisting of mitochondrial citrate synthase, a mitochondrial citrate transporter, and cytosolic ATP-dependent citrate lyase, for export of acetyl units from their mitochondria (48–50). Conversion of mitochondrial acetyl-CoA to acetate, followed by its export and cytosolic ATP-dependent activation to acetyl-CoA, occurs in *Trypanosoma brucei* (51). The latter mechanism also supports slow growth of pyruvate decarboxylase-negative *S. cerevisiae* mutants, which cannot use the PDH bypass for cytosolic acetyl-CoA synthesis (52). The ATP requirement of these naturally occurring acetyl-CoA shuttles is consistent with our hypothesis that *in vivo* concentrations of acetyl-CoA in cytosol and mitochondria of wild-type yeast cells do not allow outward translocation of acetyl units via the energy-independent carnitine shuttle. Quantification of trade-offs between ATP efficiency and *in vivo* kinetics of cytosolic acetyl-CoA provision via different pathways requires analysis of mitochondrial and cytosolic acetyl-CoA pools in wild-type and engineered strains*.* Such studies will, however, have to await development of techniques for accurate measurement of acetyl-CoA concentrations in different cellular compartments.

*YAT2*, the third gene in which a point mutation stimulated carnitine-dependent growth of *acs1*Δ *acs2*Δ strains, was reported to encode a carnitine acetyltransferase (15). Yat2 shows substantial sequence identity with the two other yeast carnitine acetyltransferases (28% and 22% amino acid sequence identity with Yat1 and Cat2, respectively [53]). However, Yat2 is substantially longer than Yat1 and Cat2, by 236 and 253 amino acids, respectively, and its 169-amino-acid C-terminal sequence is conserved only in some closely related orthologs within the *Saccharomycetaceae* (54). The mutation in *YAT2* is intriguing because Cat2 (active in the mitochondrial and peroxisomal matrices) and Yat1 (active in the cytosol) should in theory suffice to form a functional mitochondrial carnitine shuttle. Prompted by its essential role in reversal of the mitochondrial carnitine shuttle in evolved strain IMS0482, we sought to compare enzyme kinetics of wild-type Yat2 and Yat2^P58R^. Our inability to detect activity of either Yat2 isoform in cell extracts does not rule out the possibility that these proteins are carnitine acetyltransferases. Combined with the impact of a mutation in *YAT2* on *in vivo* carnitine shuttle activity, this result underlines the need for further biochemical characterization of Yat2.

### (Energetic) implications of the carnitine shuttle in cytosolic acetyl-CoA provision for biotechnological applications.

In the native *S. cerevisiae* pathway for cytosolic acetyl-CoA synthesis, cytosolic acetate is activated by the Acs1 and/or Acs2 acetyl-CoA synthetases (2, 26, 55, 56). This activation involves hydrolysis of ATP to AMP and pyrophosphate which, when pyrophosphate is subsequently hydrolyzed to inorganic phosphate, is equivalent to the hydrolysis of 2 mol of ATP to ADP and inorganic phosphate. Cytosolic acetyl-CoA is an important precursor for many industrially relevant compounds, and much effort has been invested in metabolic engineering of alternative, more-ATP-efficient pathways for cytosolic acetyl-CoA supply into *S. cerevisiae*. Examples of such strategies include cytosolic expression of heterologous phosphoketolase and phosphotransacetylase, acetylating acetaldehyde dehydrogenase, pyruvate-formate lyase, and a heterologous pyruvate dehydrogenase complex (31, 57, 58). The present study demonstrates that reversal of the mitochondrial carnitine shuttle can directly link acetyl-CoA synthesis via the mitochondrial PDH complex, the predominant source of acetyl-CoA in aerobic, glucose-grown *S. cerevisiae* cultures (36), to provision of cytosolic acetyl-CoA. The low specific growth rates of the evolved and reverse engineered l-carnitine-dependent strains indicate that this novel strategy for engineering cytosolic acetyl-CoA provision in *S. cerevisiae* requires optimization before industrial implementation can be considered. Progress in this direction would provide a strong incentive to engineer a complete l-carnitine biosynthesis pathway in *S. cerevisiae*. Despite recent advances (59), synthesis of the key precursor trimethyl-lysine in *S. cerevisiae* remains an important metabolic engineering challenge.

Export of acetyl units from mitochondria via the carnitine shuttle may also be relevant for eukaryotic cell factories other than *S. cerevisiae*. Oleaginous eukaryotes such as the yeast *Yarrowia lipolytica* employ the mitochondrial PDH complex and a citrate-oxaloacetate shuttle to provide cytosolic acetyl-CoA for lipid synthesis (49, 60). The citrate-oxaloacetate shuttle requires 1 ATP for each molecule of mitochondrial pyruvate converted into cytosolic acetyl-CoA. Eliminating this ATP requirement could further improve the ATP efficiency of lipid synthesis and, consequently, the lipid yield in oleaginous eukaryotes.

### Outlook.

By demonstrating *in vivo* reversibility of the mitochondrial carnitine shuttle, a ubiquitous mechanism in eukaryotes, this study provides new leads for investigating and understanding the role of this shuttle in yeast and other eukaryotes. The “switchable” l-carnitine-dependent yeast strains described here provide valuable experimental platforms for functional analysis of the native yeast carnitine shuttle, for heterologous complementation studies on carnitine shuttle components from other eukaryotes, and for engineering of a complete l-carnitine biosynthesis pathway into *S. cerevisiae* (59). After further optimization of the kinetics, the “reverse” mitochondrial carnitine shuttle offers a potential new strategy for energetically efficient synthesis of cytosolic acetyl-CoA as a precursor for a wide range of biotechnologically relevant compounds by eukaryotic cell factories.

## MATERIALS AND METHODS

### Growth media.

Yeast extract-peptone (YP) medium contained 10 g ⋅ liter^−1^ Bacto yeast extract (BD, Franklin Lakes, NJ, USA) and 20 g ⋅ liter^−1^ Bacto peptone (BD) in demineralized water. Synthetic medium with ammonium as the nitrogen source (SM-ammonium) was prepared by the method of Verduyn et al. (61). Synthetic medium with urea as the nitrogen source (SM-urea) contained 38 mM urea and 38 mM K_2_SO_4_ instead of (NH_4_)_2_SO_4_. SM-ammonium was autoclaved at 121°C for 20 min, and SM-urea was sterilized using 0.2-µm bottle-top filters (Thermo Fisher Scientific, Waltham, MA, USA). Solid media were prepared by the addition of 20 g ⋅ liter^−1^ agar (BD), prior to autoclaving at 121°C for 20 min. Where indicated, urea was added after heat sterilization of the solid media from a filter-sterilized 100-fold-concentrated stock solution.

### Strains, growth conditions, and storage.

All *S. cerevisiae* strains used in this study (Table 1) share the CEN.PK genetic background (62, 63). Shake flask cultures in 500-ml flasks with 100 ml SM-urea and 20 g ⋅ liter^−1^ glucose were grown at 30°C in an Innova incubator shaker (New Brunswick Scientific, Edison, NJ, USA) set at 200 rpm. Stock cultures were grown in YP medium with 20 g ⋅ liter^−1^ glucose. Where indicated, lipoic acid was added to sterile media to a concentration of 50 ng ⋅ liter^−1^. A 50-mg ⋅ liter^−1^ stock solution of lipoic acid was prepared by dissolving 5 g ⋅ liter^−1^ (±)-α-lipoic acid (Sigma-Aldrich, St. Louis, MO, USA) in ethanol and diluting the resulting solution 100-fold in sterile demineralized water. l-Carnitine (Sigma-Aldrich) was added to sterile media from a 40-g ⋅ liter^−1^ filter-sterilized stock solution at the concentration indicated. Frozen stock cultures of yeast strains were prepared by adding glycerol (30%, vol/vol) to exponentially growing shake flask cultures and freezing 1-ml aliquots at −80°C.

### Plasmid construction.

Guide RNA (gRNA) plasmids for clustered regularly interspaced short palindromic repeat (CRISPR)/Cas9-based genome editing (see Table S1 in the supplemental material) were constructed as described previously (33). In short, double-gRNA cassettes were PCR amplified using the primer(s) indicated in Tables S1 and S2. Plasmid backbones containing the desired marker gene were obtained by PCR with primer 6005, using the appropriate pROS plasmid (Table S1) as a template. The two fragments were then assembled into a plasmid with the Gibson Assembly kit (New England Biolabs, Ipswich, MA, USA) or NEBuilder HiFi DNA assembly cloning kit (New England Biolabs). Multicopy plasmids carrying wild-type *YAT2* and mutated *YAT2* variants were based on the pRS426 expression vector (64). *pADH1-YAT2-tYAT2* and *pADH1-YAT2^C173G^*-tYAT2 fragments were PCR amplified from strains IMX745 and IMS0482, respectively, using primers 8902 and 8903 (sequences of these cassettes are presented in Table S3) and then inserted into the EcoRI-XhoI-linearized pRS426 backbone with the NEBuilder HiFi DNA assembly cloning kit. After transforming the resulting plasmids to *Escherichia coli* and confirmation of their DNA sequences by Illumina sequencing, this yielded pUDE390 (2µm ori *URA3 pADH1-YAT2-tYAT2*) and pUDE391 (2µm ori *URA3 pADH1-YAT2^C173G^*-tYAT2). A multicopy plasmid carrying the *CAT2* gene under control of the *TDH3* promoter was similarly obtained by assembling a pRS426 backbone with a *CAT2* PCR fragment using the Gibson Assembly kit. The *TDH3* promoter and *CYC1* terminator sequences were synthesized and assembled into the pRS426 vector by GenScript (Piscataway, NJ, USA). The resulting plasmid was linearized by PCR amplification using primers 3627 and 3921. The *CAT2* open reading frame (ORF) was amplified via PCR from *S*. *cerevisiae* CEN.PK113-7D genomic DNA using primers 5948 and 5949. Gibson Assembly of the two fragments yielded pUDE336 (2µm ori *URA3 pTDH3-CAT2-His_6_-tCYC1*). The DNA sequence of the *pTDH3-CAT2-His_6_-tCYC1* cassette is presented in Table S3.

### Strain construction.

*S. cerevisiae* strains were transformed by the method of Gietz and Woods (65), and transformants were selected on solid YP medium with 20 g ⋅ liter^−1^ glucose. Appropriate antibiotics were added at the following concentrations: G418 (InvivoGen, San Diego, CA, USA), 200 mg ⋅ liter^−1^; hygromycin B (InvivoGen), 200 mg ⋅ liter^−1^; nourseothricin (Jena Bioscience, Jena, Germany), 100 mg ⋅ liter^−1^. Lipoic acid was added as indicated above. Throughout the text we refer to chromosomally integrated gene clusters with four-capital acronyms surrounded by curly brackets (based on the common practice in set theory for indicating a collection of elements). A mutation in a gene that is part of the cluster is indicated within the curly brackets. For example, {CARN,*YAT2^C173G^*} refers to the {CARN} set in which the *YAT2* gene carries a C173G nucleotide change.

Unless indicated otherwise, genetic engineering was done using CRISPR/Cas9 (33). The platform strain with constitutive expression of the genes involved in the carnitine shuttle (*HNM1*, *AGP2*, *CRC1*, *YAT1*, *YAT2*, and *CAT2*) was constructed by modification of the previously constructed strain IMX719 (33), which had *ACS1* and *ACS2* replaced by the genes required for an active, lipoylated cytosolic *Enterococcus faecalis* PDH complex {PDHL}. Analogous to a previous description (8), the genes involved in the carnitine shuttle were placed under the control of strong constitutive promoters and integrated into the *SGA1* locus of strain IMX719, resulting in strain IMX745 (*acs1*Δ *acs2*Δ::{PDHL} *sga1*Δ::{CARN}) (Table 1). To remove the *E. faecalis* PDH genes {PDHL} or the set of carnitine shuttle expression cassettes {CARN} from strains IMS0482 and IMS0483, either plasmid pUDR072 (to remove {PDHL}) or pUDR073 (to remove {CARN}) was transformed together with a repair fragment obtained by annealing oligonucleotides 7349 and 7350 or oligonucleotides 8012 and 8013 (see Table S2 in the supplemental material), respectively, resulting in strains IMW074 to IMW077. Deletion of *PDA1* and *ACH1* in strains IMS0482 and IMS0483 was done by transformation with pUDR047 (with oligonucleotides 6157 and 6158) and pUDR085 (with oligonucleotides 6160 and 6161), resulting in strains IMW078 to IMW082. To introduce the *MCT1^T641G^* mutation, plasmid pUDR080 and a repair fragment obtained by annealing oligonucleotides 8417 and 8418 was transformed into strain IMX745 (Table 1), resulting in strain IMX847. Similarly, the *RTG2^G503T^* mutation was introduced in strain IMX745 by transforming plasmid pUDR078 and oligonucleotides 8430 and 8431, resulting in strain IMX849. The *MCT1^T641G^ RTG2^G503T^* double mutations were introduced in strain IMX745 using plasmid pUDR079 using oligonucleotides 8417, 8418, 8430, and 8431, resulting in strain IMX852. To selectively introduce the *YAT2^C173G^* mutation in the *ADH1* promoter-driven gene, not in the *YAT2*-promoter driven gene (at chromosome V), the single-nucleotide polymorphism (SNP) was introduced in {CARN} via a two-step strategy. First, a synthetic CRISPR target site was introduced by transformation of strains IMX745, IMX847, IMX849, and IMX852 with plasmid pUDR073 and oligonucleotides 8621 and 8622, thereby removing part of the *ADH1* promoter and part of the *YAT2* ORF. Next, the fragment containing the *YAT2^C173G^* mutation was PCR amplified from the IMS0482 genome using primers 8618 and 8619 and cotransformed with plasmid pUDR105, introducing the *YAT2^C173G^* mutation and resulting in strains IMX907, IMX909, IMX911, and IMX913. In all these cases, after introduction of the desired mutations, the double-gRNA plasmids were removed, followed by confirmation of the SNPs by Sanger sequencing (BaseClear BV, Leiden, The Netherlands) using the primers indicated in Table S2. The ORFs of *YAT2* (the copy present in {CARN}), *RTG2*, and *MCT1* were deleted from the genomes of strains IMX852, IMX909, and IMX911, respectively, by transforming the following plasmids and repair fragments: for strain IMX852, plasmid pUDR073 and oligonucleotides 8874 and 8875; for strain IMX909, plasmid pUDR078 and oligonucleotides 8428 and 8429; and for strain IMX911, plasmid pUDR080 and oligonucleotides 8415 and 8416. After gene knockout was confirmed by diagnostic PCR (Table S2), the resulting strains were named IMX932 to IMX934, respectively.

The *pADH1-YAT2-tYAT2* variants were integrated in the *cas9*-bearing reference strain IMX585. *pADH1-YAT2-tYAT2* (wild-type) and *pADH1-YAT2^C173G^*-tYAT2 cassettes were amplified with PCR using primers 8647 and 8648 from genomic DNA of strains IMX745 and IMS0482, respectively. The resulting cassettes had overlaps with the promoter and terminator of *SGA1*, enabling integration into the *SGA1* locus. Cas9 was directed to the *SGA1* locus using the gRNA plasmid pUDR119 (see Table S1 in the supplemental material), following integration of the cassette by *in vivo* homologous recombination. After confirmation of correct integration and sequence by PCR and Sanger sequencing, plasmid pUDR119 was removed as described earlier (33), resulting in strains IMX923 and IMX925, respectively. To obtain the multicopy-based *YAT2-* and *CAT2*-expressing strains, plasmids pUDE336, pUDE390, and pUDE391 were transformed to strain CEN.PK113-5D, resulting in strains IME233, IME320, and IME321, respectively (Table 1).

To obtain strain CEN.PK215-4A (*cat2*Δ *yat1*Δ), *CAT2* and *YAT1* were deleted by transformation of a *kanMX* marker cassette, obtained by PCR using pUG6 as the template (66) and primers 9237 and 9238 for the *CAT2* deletion cassette and primers 9239 and 9240 for the *YAT1* deletion cassette. The amplified *kanMX* cassettes were used as selectable markers to replace the target genes in the prototrophic diploid strain CEN.PK122. Transformants were verified for correct gene replacement by diagnostic PCR (see Table S2 in the supplemental material). After sporulation and tetrad dissection, the corresponding haploid deletion strains, CEN.PK194-2C (*MAT***a** *cat2*Δ) and CEN.PK196-2C (*MAT*α *yat1*Δ), were obtained. To obtain a strain with both *CAT2* and *YAT1* deleted, strains CEN.PK194-2C and CEN.PK196-2C were crossed. After tetrad dissection, spores were subsequently analyzed by diagnostic PCR to confirm correct deletion of both genes, resulting in strain CEN.PK215-4A (*cat2*Δ *yat1*Δ) (Table 1).

### Molecular biology techniques.

PCR amplification with the Phusion Hot Start II high-fidelity polymerase (Thermo Fisher Scientific) was performed according to the manufacturer’s instructions, using high-performance liquid chromatography (HPLC)- or polyacrylamide gel electrophoresis (PAGE)-purified oligonucleotide primers (Sigma-Aldrich). Diagnostic colony PCR was performed on randomly picked transformed colonies, using DreamTaq (Thermo Fisher Scientific) and desalted primers (Sigma-Aldrich). DNA fragments obtained by PCR were separated by gel electrophoresis on 1% (wt/vol) agarose gels (Thermo Fisher Scientific) in TAE (Tris-acetate-EDTA) buffer (Thermo Fisher Scientific). Alternatively, fragments were purified using the GenElute PCR cleanup kit (Sigma-Aldrich). Plasmids were isolated from *E. coli* with Sigma GenElute plasmid kit (Sigma-Aldrich) according to the supplier’s manual. Yeast genomic DNA was isolated using a YeaStar genomic DNA kit (Zymo Research) or using a sodium dodecyl sulfate/lithium acetate-based lysis protocol (67). *E. coli* XL1-Blue (GE Healthcare Life Sciences, The Netherlands) was used for chemical transformation or for electroporation. Chemical transformation was conducted by the method of Inoue et al. (68). Electroporation was performed in a 2-mm cuvette (catalog no. 1652086; Bio-Rad, Hercules, CA, USA) using a Gene Pulser Xcell electroporation system (Bio-Rad), following the manufacturer’s protocol. Electrocompetent *E. coli* cells were prepared according to the same protocol, with the exception that, during preparation of competent cells, *E. coli* was grown in LB medium without sodium chloride.

### Laboratory evolution.

Strain IMX745 was inoculated in 500-ml shake flasks containing 100 ml SM-urea with 20 g ⋅ liter^−1^ glucose and 400 mg ⋅ liter^−1^ l-carnitine. When stationary phase was reached, 1 to 3 ml of culture was transferred to a new shake flask. After six or seven serial shake flask transfers, eight individual cells were isolated from each evolution experiment using a micromanipulator (Singer Instruments, Watchet, United Kingdom) and placed on SM-urea plates with 20 g ⋅ liter^−1^ glucose and 400 mg ⋅ liter^−1^ l-carnitine. For each evolution experiment, one colony was selected and restreaked once, yielding strains IMS0482 (evolution line 1) and IMS0483 (evolution line 2) (Table 1).

### DNA sequencing and sequence analysis.

After isolation of genomic DNA (69) from strains IMX745, IMS0482, and IMS0483, 350-bp insert libraries were constructed and paired-end sequenced (100-bp reads) with an Illumina HiSeq 2500 sequencer (Baseclear BV, Leiden, The Netherlands). At least 500 Mb of sequence data, corresponding to a ca. 40-fold coverage, was generated for each strain. Plasmids pUDE390 and pUDE391 were sequenced in-house using the Illumina MiSeq platform (San Diego, CA, USA). After quantification of plasmid DNA with the Qubit 2.0 fluorometer (Thermo Fisher Scientific), DNA libraries were prepared using the Nextera XT DNA kit (Illumina). Paired-end reads (300 bp) of plasmid DNA generated on the MiSeq platform were mapped to an *in silico*-generated plasmid sequence using the Burrows-Wheeler alignment tool (70) and processed with Pilon (71). Sequence reads of genomic DNA were mapped onto the CEN.PK113-7D genome (63), supplemented with sequences containing the modified *SGA1*, *ACS2*, and *CAN1* loci, using the Burrows-Wheeler alignment tool (70). Data were further processed with Pilon (71), and sequence variations were extracted from the Pilon output file “.changes.” The uniqueness of sequence differences in strains IMS0482 and IMS0483 was manually confirmed by comparison with strain IMX745 using the Integrative Genomics Viewer (72). Copy number variations in strains IMS0482 and IMS0483, relative to strain IMX745, were determined with the Poisson mixture model-based algorithm Magnolya (37). 

### Growth studies in shake flasks and using spot plate assays.

For growth studies in shake flasks and using spot plates, strains were pregrown in shake flasks with SM-urea and 20 g ⋅ liter^−1^ glucose with lipoic acid or l-carnitine, where appropriate. For growth studies in shake flasks, cells were washed twice with synthetic medium (61) and transferred to new shake flasks with SM-urea containing 20 g ⋅ liter^−1^ glucose and 40 mg ⋅ liter^−1^ l-carnitine or 50 ng ⋅ liter^−1^ lipoic acid, where indicated. Growth rates were based on optical density at 660 nm (OD_660_) measurements using a Libra S11 spectrophotometer (Biochrom, Cambridge, United Kingdom). Culture viability was estimated with the FungaLight AM-CFDA (acetoxymethyl ester 5-carboxyfluorescein diacetate)/propidium iodide yeast viability kit (Invitrogen, Carlsbad, CA) and a Cell Lab Quanta SC MPL flow cytometer (Beckman Coulter, Woerden, The Netherlands) as described previously (73). For the preparation of spot plates, precultures were washed once with synthetic medium and diluted in synthetic medium to an OD_660_ of 0.273 (corresponding to 2 × 10^6^ cells ⋅ ml^−1^). Five-microliter samples of a dilution series, containing an estimated 2 × 10^5^, 2 × 10^4^, and 2 × 10^3^ cells per ml, were spotted on SM-urea agar plates with 20 g ⋅ liter^−1^ glucose and l-carnitine (400 mg ⋅ liter^−1^) or lipoic acid (50 ng ⋅ liter^−1^) as indicated.

### Enzyme activity assays.

Cell extracts were prepared as described before (8) from mid-exponentially growing cultures. The growth medium was SM-ammonium with either 20 g ⋅ liter^−1^ glucose or 2% (vol/vol) ethanol as the carbon source and, where required, lipoic acid. Activities in cell extracts of carnitine acetyltransferase activity (8) and glucose-6-phosphate dehydrogenase (74) (the latter activity was used to verify the quality of cell extracts) were assayed spectrophotometrically as described previously (8). Protein concentrations in cell extracts were determined by the Lowry method (75).

### Nucleotide sequence accession number.

Raw sequencing data of strains IMX745, IMS0482, and IMS0483 are deposited at the NCBI Sequence Read Archive (http://www.ncbi.nlm.nih.gov/sra) under BioProject identifier (ID) or accession number PRJNA313402.

## SUPPLEMENTAL MATERIAL

Data Set S1  Original, annotated photographs of spot plates used in Fig. 3, Fig. 4, Fig. 5, and Fig. 6. As spot plate assays were done in duplicate, for each medium, the photos show two plates per strain. Download Data Set S1, PDF file, 1 MB

Table S1  Guide RNA plasmids used in this study. For amplification of the double-guide RNA cassette, a pROS plasmid (33) was used as the template with the primer(s) indicated. Table S1, DOCX file, 0.04 MB

Table S2  Primers used in this study. Table S2, DOCX file, 0.04 MB

Table S3  Sequences of the *YAT2* and *CAT2* gene cassettes used in this study. The sequences of the open reading frames are underlined, and the C173G mutation is indicated in red. Table S3, DOCX file, 0.04 MB

## References

[1] Pokholok DK, Harbison CT, Levine S, Cole M, Hannett NM, Lee TI, Bell GW, Walker K, Rolfe PA, Herbolsheimer E, Zeitlinger J, Lewitter F, Gifford DK, Young RA 2005 Genome-wide map of nucleosome acetylation and methylation in yeast. Cell 122:517–527. doi:10.1016/j.cell.2005.06.026. 16122420

[2] Takahashi H, McCaffery JM, Irizarry RA, Boeke JD 2006 Nucleocytosolic acetyl-coenzyme A synthetase is required for histone acetylation and global transcription. Mol Cell 23:207–217. doi:10.1016/j.molcel.2006.05.040. 16857587

[3] Galdieri L, Zhang T, Rogerson D, Lleshi R, Vancura A 2014 Protein acetylation and acetyl coenzyme A metabolism in budding yeast. Eukaryot Cell 13:1472–1483. doi:10.1128/EC.00189-14. 25326522 PMC4248685

[4] Nielsen J 2014 Synthetic biology for engineering acetyl coenzyme A metabolism in yeast. mBio 5:e00520-16. doi:10.1128/mBio.02153-14. PMC4222110 25370498

[5] Nielsen J, Larsson C, Van Maris AJA, Pronk JT 2013 Metabolic engineering of yeast for production of fuels and chemicals. Curr Opin Biotechnol 24:398–404. doi:10.1016/j.copbio.2013.03.023. 23611565

[6] Szutowicz A, Bielarczyk H, Ronowska A, Gul-Hinc S, Klimaszewska-Łata J, Dyś A, Zyśk M, Pawełczyk T 2014 Intracellular redistribution of acetyl-CoA, the pivotal point in differential susceptibility of cholinergic neurons and glial cells to neurodegenerative signals. Biochem Soc Trans 42:1101–1106. doi:10.1042/BST20140078. 25110009

[7] Strijbis K, Distel B 2010 Intracellular acetyl unit transport in fungal carbon metabolism. Eukaryot Cell 9:1809–1815. doi:10.1128/EC.00172-10. 20889721 PMC3008284

[8] Van Rossum HM, Kozak BU, Niemeijer MS, Duine HJ, Luttik MAH, Boer VM, Kötter P, Daran J-MG, Van Maris AJA, Pronk JT 2016 Alternative reactions at the interface of glycolysis and citric acid cycle in *Saccharomyces cerevisiae*. FEMS Yeast Res 16:fow017. doi:10.1093/femsyr/fow017. 26895788 PMC5815053

[9] Van Roermund CW, Elgersma Y, Singh N, Wanders RJ, Tabak HF 1995 The membrane of peroxisomes in *Saccharomyces cerevisiae* is impermeable to NAD(H) and acetyl-CoA under *in vivo* conditions. EMBO J 14:3480–3486. 7628449 10.1002/j.1460-2075.1995.tb07354.x PMC394415

[10] Fukui S, Tanaka A 1979 Yeast peroxisomes. Trends Biochem Sci 4:246–249. doi:10.1016/0968-0004(79)90214-7.

[11] Bieber LL 1988 Carnitine. Annu Rev Biochem 57:261–283. doi:10.1146/annurev.bi.57.070188.001401. 3052273

[12] Hiltunen JK, Mursula AM, Rottensteiner H, Wierenga RK, Kastaniotis AJ, Gurvitz A 2003 The biochemistry of peroxisomal β-oxidation in the yeast *Saccharomyces cerevisiae*. FEMS Microbiol Rev 27:35–64. doi:10.1016/S0168-6445(03)00017-2. 12697341

[13] Vaz FM, Wanders RJA 2002 Carnitine biosynthesis in mammals. Biochem J 361:417–429. doi:10.1042/bj3610417. 11802770 PMC1222323

[14] Strijbis K, Van Roermund CWT, Hardy GP, Van den Burg J, Bloem K, De Haan J, Van Vlies N, Wanders RJA, Vaz FM, Distel B 2009 Identification and characterization of a complete carnitine biosynthesis pathway in *Candida albicans*. FASEB J 23:2349–2359. doi:10.1096/fj.08-127985. 19289605

[15] Swiegers JH, Dippenaar N, Pretorius IS, Bauer FF 2001 Carnitine-dependent metabolic activities in *Saccharomyces cerevisiae*: three carnitine acetyltransferases are essential in a carnitine-dependent strain. Yeast 18:585–595. doi:10.1002/yea.712. 11329169

[16] Aouida M, Rubio-Texeira M, Thevelein JM, Poulin R, Ramotar D 2013 Agp2, a member of the yeast amino acid permease family, positively regulates polyamine transport at the transcriptional level. PLoS One 8:e65717. doi:10.1371/journal.pone.0065717. 23755272 PMC3670898

[17] Van Roermund CW, Hettema EH, Van den Berg M, Tabak HF, Wanders RJ 1999 Molecular characterization of carnitine-dependent transport of acetyl-CoA from peroxisomes to mitochondria in *Saccharomyces cerevisiae* and identification of a plasma membrane carnitine transporter, Agp2p. EMBO J 18:5843–5852. doi:10.1093/emboj/18.21.5843. 10545096 PMC1171650

[18] Elgersma Y, Van Roermund CW, Wanders RJ, Tabak HF 1995 Peroxisomal and mitochondrial carnitine acetyltransferases of *Saccharomyces cerevisiae* are encoded by a single gene. EMBO J 14:3472–3479. 7628448 10.1002/j.1460-2075.1995.tb07353.x PMC394414

[19] Schmalix W, Bandlow W 1993 The ethanol-inducible *YAT1* gene from yeast encodes a presumptive mitochondrial outer carnitine acetyltransferase. J Biol Chem 268:27428–27439. 8262985

[20] Huh W-K, Falvo JV, Gerke LC, Carroll AS, Howson RW, Weissman JS, O’Shea EK 2003 Global analysis of protein localization in budding yeast. Nature 425:686–691. doi:10.1038/nature02026. 14562095

[21] Koh JLY, Chong YT, Friesen H, Moses A, Boone C, Andrews BJ, Moffat J 2015 CYCLoPs: a comprehensive database constructed from automated analysis of protein abundance and subcellular localization patterns in *Saccharomyces cerevisiae*. G3 (Bethesda) 5:1223–1232. doi:10.1534/g3.115.017830. 26048563 PMC4478550

[22] Kohlhaw GB, Tan-Wilson A 1977 Carnitine acetyltransferase: candidate for the transfer of acetyl groups through the mitochondrial membrane of yeast. J Bacteriol 129:1159–1161. 320182 10.1128/jb.129.2.1159-1161.1977 PMC235061

[23] Palmieri L, Lasorsa FM, Iacobazzi V, Runswick MJ, Palmieri F, Walker JE 1999 Identification of the mitochondrial carnitine carrier in *Saccharomyces cerevisiae*. FEBS Lett 462:472–476. doi:10.1016/S0014-5793(99)01555-0. 10622748

[24] Franken J, Kroppenstedt S, Swiegers JH, Bauer FF 2008 Carnitine and carnitine acetyltransferases in the yeast *Saccharomyces cerevisiae*: a role for carnitine in stress protection. Curr Genet 53:347–360. doi:10.1007/s00294-008-0191-0. 18427809

[25] Grunau S, Mindthoff S, Rottensteiner H, Sormunen RT, Hiltunen JK, Erdmann R, Antonenkov VD 2009 Channel-forming activities of peroxisomal membrane proteins from the yeast *Saccharomyces cerevisiae*. FEBS J 276:1698–1708. doi:10.1111/j.1742-4658.2009.06903.x. 19220856

[26] Pronk JT, Yde Steensma H, Van Dijken JP 1996 Pyruvate metabolism in *Saccharomyces cerevisiae*. Yeast 12:1607–1633. doi:10.1002/(SICI)1097-0061(199612)12:16 &lt;1607::AID-YEA70&gt;3.0.CO;2-4. 9123965

[27] Flamholz A, Noor E, Bar-Even A, Milo R 2012 eQuilibrator—the biochemical thermodynamics calculator. Nucleic Acids Res 40:D770–D775. doi:10.1093/nar/gkr874. 22064852 PMC3245061

[28] Holzer H, Goedde HW 1957 Two ways from pyruvate to acetyl-coenzyme A in yeast. Biochem Z 329:175–191. (In German.) 13522696

[29] Kispal G, Cseko J, Alkonyi I, Sandor A 1991 Isolation and characterization of carnitine acetyltransferase from *S. cerevisiae*. Biochim Biophys Acta 1085:217–222. doi:10.1016/0005-2760(91)90097-2. 1892891

[30] Van Maris AJA, Luttik MAH, Winkler AA, Van Dijken JP, Pronk JT 2003 Overproduction of threonine aldolase circumvents the biosynthetic role of pyruvate decarboxylase in glucose-limited chemostat cultures of *Saccharomyces cerevisiae*. Appl Environ Microbiol 69:2094–2099. doi:10.1128/AEM.69.4.2094-2099.2003. 12676688 PMC154831

[31] Kozak BU, Van Rossum HM, Luttik MAH, Akeroyd M, Benjamin KR, Wu L, De Vries S, Daran J-M, Pronk JT, Van Maris AJA 2014 Engineering acetyl coenzyme A supply: functional expression of a bacterial pyruvate dehydrogenase complex in the cytosol of *Saccharomyces cerevisiae*. mBio 5:e00520-16. doi:10.1128/mBio.01696-14. PMC4212835 25336454

[32] Knijnenburg TA, Daran J-MG, Van den Broek MA, Daran-Lapujade PAS, De Winde JH, Pronk JT, Reinders MJT, Wessels LFA 2009 Combinatorial effects of environmental parameters on transcriptional regulation in *Saccharomyces cerevisiae*: a quantitative analysis of a compendium of chemostat-based transcriptome data. BMC Genomics 10:53. doi:10.1186/1471-2164-10-53. 19173729 PMC2640415

[33] Mans R, Van Rossum HM, Wijsman M, Backx A, Kuijpers NGA, Van den Broek M, Daran-Lapujade P, Pronk JT, Van Maris AJA, Daran J-MG 2015 CRISPR/Cas9: a molecular Swiss army knife for simultaneous introduction of multiple genetic modifications in Saccharomyces cerevisiae. FEMS Yeast Res 15:fov004. doi:10.1093/femsyr/fov004. 25743786 PMC4399441

[34] Fleck CB, Brock M 2009 Re-characterisation of *Saccharomyces cerevisiae* Ach1p: fungal CoA-transferases are involved in acetic acid detoxification. Fungal Genet Biol 46:473–485. doi:10.1016/j.fgb.2009.03.004. 19298859

[35] Wenzel TJ, Van den Berg MA, Visser W, Van den Berg JA, Steensma HY 1992 Characterization of *Saccharomyces cerevisiae* mutants lacking the E1 alpha subunit of the pyruvate dehydrogenase complex. Eur J Biochem 209:697–705. doi:10.1111/j.1432-1033.1992.tb17338.x. 1330555

[36] Pronk JT, Wenzel TJ, Luttik MA, Klaassen CC, Scheffers WA, Steensma HY, Van Dijken JP 1994 Energetic aspects of glucose metabolism in a pyruvate-dehydrogenase-negative mutant of *Saccharomyces cerevisiae*. Microbiology 140:601–610. doi:10.1099/00221287-140-3-601. 8012582

[37] Nijkamp JF, Van Den Broek MA, Geertman JMA, Reinders MJT, Daran JMG, De Ridder D 2012 *De novo* detection of copy number variation by co-assembly. Bioinformatics 28:3195–3202. doi:10.1093/bioinformatics/bts601. 23047563

[38] Schneider R, Brors B, Bürger F, Camrath S, Weiss H 1997 Two genes of the putative mitochondrial fatty acid synthase in the genome of *Saccharomyces cerevisiae*. Curr Genet 32:384–388. doi:10.1007/s002940050292. 9388293

[39] Reinders J, Zahedi RP, Pfanner N, Meisinger C, Sickmann A 2006 Toward the complete yeast mitochondrial proteome: multidimensional separation techniques for mitochondrial proteomics. J Proteome Res 5:1543–1554. doi:10.1021/pr050477f. 16823961

[40] Liao X, Butow RA 1993 *RTG1* and *RTG2*: two yeast genes required for a novel path of communication from mitochondria to the nucleus. Cell 72:61–71. doi:10.1016/0092-8674(93)90050-Z. 8422683

[41] Small WC, Brodeur RD, Sandor A, Fedorova N, Li G, Butow RA, Srere PA 1995 Enzymatic and metabolic studies on retrograde regulation mutants of yeast. Biochemistry 34:5569–5576. doi:10.1021/bi00016a031. 7727418

[42] Hoja U, Marthol S, Hofmann J, Stegner S, Schulz R, Meier S, Greiner E, Schweizer E 2004 *HFA1* encoding an organelle-specific acetyl-CoA carboxylase controls mitochondrial fatty acid synthesis in *Saccharomyces cerevisiae*. J Biol Chem 279:21779–21786. doi:10.1074/jbc.M401071200. 14761959

[43] Kaushik VK, Kavana M, Volz JM, Weldon SC, Hanrahan S, Xu J, Caplan SL, Hubbard BK 2009 Characterization of recombinant human acetyl-CoA carboxylase-2 steady-state kinetics. Biochim Biophys Acta 1794:961–967. doi:10.1016/j.bbapap.2009.02.004. 19236960

[44] Chuakrut S, Arai H, Ishii M, Igarashi Y 2003 Characterization of a bifunctional archaeal acyl coenzyme A carboxylase. J Bacteriol 185:938–947. doi:10.1128/JB.185.3.938-947.2003. 12533469 PMC142822

[45] Pasquali M, Monsen G, Richardson L, Alston M, Longo N 2006 Biochemical findings in common inborn errors of metabolism. Am J Med Genet C Semin Med Genet 142C:64–76. doi:10.1002/ajmg.c.30086. 16602099

[46] Violante S, IJlst L, Te Brinke H, Tavares De Almeida I, Wanders RJA, Ventura FV, Houten SM 2013 Carnitine palmitoyltransferase 2 and carnitine/acylcarnitine translocase are involved in the mitochondrial synthesis and export of acylcarnitines. FASEB J 27:2039–2044. doi:10.1096/fj.12-216689. 23322164

[47] IJlst L, van Roermund CW, Iacobazzi V, Oostheim W, Ruiter JP, Williams JC, Palmieri F, Wanders RJ 2001 Functional analysis of mutant human carnitine acylcarnitine translocases in yeast. Biochem Biophys Res Commun 280:700–706. doi:10.1006/bbrc.2000.4178. 11162577

[48] Brunengraber H, Lowenstein JM 1973 Effect of (−)-hydroxycitrate on ethanol metabolism. FEBS Lett 36:130–132. doi:10.1016/0014-5793(73)80353-9. 4754260

[49] Boulton CA, Ratledge C 1981 Correlation of lipid accumulation in yeasts with possession of ATP:citrate lyase. Microbiology 127:169–176. doi:10.1099/00221287-127-1-169.

[50] Hynes MJ, Murray SL 2010 ATP-citrate lyase is required for production of cytosolic acetyl coenzyme A and development in *Aspergillus nidulans*. Eukaryot Cell 9:1039–1048. doi:10.1128/EC.00080-10. 20495057 PMC2901662

[51] Rivière L, Moreau P, Allmann S, Hahn M, Biran M, Plazolles N, Franconi JM, Boshart M, Bringaud F 2009 Acetate produced in the mitochondrion is the essential precursor for lipid biosynthesis in procyclic trypanosomes. Proc Natl Acad Sci U S A 106:12694–12699. doi:10.1073/pnas.0903355106. 19625628 PMC2722340

[52] Chen Y, Zhang Y, Siewers V, Nielsen J 2015 Ach1 is involved in shuttling mitochondrial acetyl units for cytosolic C2 provision in *Saccharomyces cerevisiae* lacking pyruvate decarboxylase. FEMS Yeast Res 15:fov015. doi:10.1093/femsyr/fov015. 25852051

[53] Wapinski I, Pfeffer A, Friedman N, Regev A 2007 Automatic genome-wide reconstruction of phylogenetic gene trees. Bioinformatics 23:i549–i558. doi:10.1093/bioinformatics/btm193. 17646342

[54] Huerta-Cepas J, Szklarczyk D, Forslund K, Cook H, Heller D, Walter MC, Rattei T, Mende DR, Sunagawa S, Kuhn M, Jensen LJ, Von Mering C, Bork P 2016 eggNOG 4.5: a hierarchical orthology framework with improved functional annotations for eukaryotic, prokaryotic and viral sequences. Nucleic Acids Res 44:D286–D293. doi:10.1093/nar/gkv1248. 26582926 PMC4702882

[55] Van den Berg MA, Steensma HY 1995 *ACS2*, a *Saccharomyces cerevisiae* gene encoding acetyl-coenzyme A synthetase, essential for growth on glucose. Eur J Biochem 231:704–713. doi:10.1111/j.1432-1033.1995.tb20751.x. 7649171

[56] De Jong-Gubbels P, Van den Berg MA, Steensma HY, Van Dijken JP, Pronk JT 1997 The *Saccharomyces cerevisiae* acetyl-coenzyme A synthetase encoded by the *ACS1* gene, but not the *ACS2*-encoded enzyme, is subject to glucose catabolite inactivation. FEMS Microbiol Lett 153:75–81. doi:10.1111/j.1574-6968.1997.tb10466.x. 9252575

[57] Sonderegger M, Schümperli M, Sauer U 2004 Metabolic engineering of a phosphoketolase pathway for pentose catabolism in *Saccharomyces cerevisiae*. Appl Environ Microbiol 70:2892–2897. doi:10.1128/AEM.70.5.2892-2897.2004. 15128548 PMC404438

[58] Kozak BU, Van Rossum HM, Benjamin KR, Wu L, Daran J-MG, Pronk JT, Van Maris AJA 2014 Replacement of the *Saccharomyces cerevisiae* acetyl-CoA synthetases by alternative pathways for cytosolic acetyl-CoA synthesis. Metab Eng 21:46–59. doi:10.1016/j.ymben.2013.11.005. 24269999

[59] Franken J, Burger A, Swiegers JH, Bauer FF 2015 Reconstruction of the carnitine biosynthesis pathway from *Neurospora crassa* in the yeast *Saccharomyces cerevisiae*. Appl Microbiol Biotechnol 99:6377–6389. doi:10.1007/s00253-015-6561-x. 25851717

[60] Liu X-Y, Chi Z-M, Liu G-L, Madzak C, Chi Z-M 2013 Both decrease in *ACL1* gene expression and increase in *ICL1* gene expression in marine-derived yeast *Yarrowia lipolytica* expressing *INU1* gene enhance citric acid production from inulin. Mar Biotechnol 15:26–36. doi:10.1007/s10126-012-9452-5. 22562483

[61] Verduyn C, Postma E, Scheffers WA, Van Dijken JP 1992 Effect of benzoic acid on metabolic fluxes in yeasts: a continuous-culture study on the regulation of respiration and alcoholic fermentation. Yeast 8:501–517. doi:10.1002/yea.320080703. 1523884

[62] Entian KD, Kötter P 2007 Yeast genetic strain and plasmid collections. Methods Microbiol 36:629–666.

[63] Nijkamp JF, Van den Broek M, Datema E, De Kok S, Bosman L, Luttik MA, Daran-Lapujade P, Vongsangnak W, Nielsen J, Heijne WHM, Klaassen P, Paddon CJ, Platt D, Kötter P, Van Ham RC, Reinders MJT, Pronk JT, De Ridder D, Daran J-M 2012 *De novo* sequencing, assembly and analysis of the genome of the laboratory strain *Saccharomyces cerevisiae* CEN.PK113-7D, a model for modern industrial biotechnology. Microb Cell Fact 11:36. doi:10.1186/1475-2859-11-36. 22448915 PMC3364882

[64] Christianson TW, Sikorski RS, Dante M, Shero JH, Hieter P 1992 Multifunctional yeast high-copy-number shuttle vectors. Gene 110:119–122. doi:10.1016/0378-1119(92)90454-W. 1544568

[65] Gietz RD, Woods RA 2002 Transformation of yeast by lithium acetate/single-stranded carrier DNA/polyethylene glycol method. Methods Enzymol 350:87–96. doi:10.1016/S0076-6879(02)50957-5. 12073338

[66] Güldener U, Heck S, Fielder T, Beinhauer J, Hegemann JH 1996 A new efficient gene disruption cassette for repeated use in budding yeast. Nucleic Acids Res 24:2519–2524. doi:10.1093/nar/24.13.2519. 8692690 PMC145975

[67] Lõoke M, Kristjuhan K, Kristjuhan A 2011 Extraction of genomic DNA from yeasts for PCR-based applications. Biotechniques 50:325–328. doi:10.2144/000113672. 21548894 PMC3182553

[68] Inoue H, Nojima H, Okayama H 1990 High efficiency transformation of *Escherichia coli* with plasmids. Gene 96:23–28. doi:10.1016/0378-1119(90)90336-P. 2265755

[69] De Kok S, Nijkamp JF, Oud B, Roque FC, Ridder D, Daran J-M, Pronk JT, Maris AJA 2012 Laboratory evolution of new lactate transporter genes in a *jen1*Δ mutant of *Saccharomyces cerevisiae* and their identification as *ADY2* alleles by whole-genome resequencing and transcriptome analysis. FEMS Yeast Res 12:359–374. doi:10.1111/j.1567-1364.2011.00787.x. 22257278

[70] Li H, Durbin R 2009 Fast and accurate short read alignment with Burrows-Wheeler transform. Bioinformatics 25:1754–1760. doi:10.1093/bioinformatics/btp324. 19451168 PMC2705234

[71] Walker BJ, Abeel T, Shea T, Priest M, Abouelliel A, Sakthikumar S, Cuomo CA, Zeng Q, Wortman J, Young SK, Earl AM 2014 Pilon: an integrated tool for comprehensive microbial variant detection and genome assembly improvement. PLoS One 9:e112963. doi:10.1371/journal.pone.0112963. 25409509 PMC4237348

[72] Thorvaldsdóttir H, Robinson JT, Mesirov JP 2013 Integrative Genomics Viewer (IGV): high-performance genomics data visualization and exploration. Brief Bioinform 14:178–192. doi:10.1093/bib/bbs017. 22517427 PMC3603213

[73] Boender LGM, Almering MJH, Dijk M, Van Maris AJA, De Winde JH, Pronk JT, Daran-Lapujade P 2011 Extreme calorie restriction and energy source starvation in *Saccharomyces cerevisiae* represent distinct physiological states. Biochim Biophys Acta 1813:2133–2144. doi:10.1016/j.bbamcr.2011.07.008. 21803078

[74] Postma E, Verduyn C, Scheffers WA, Van Dijken JP 1989 Enzymic analysis of the Crabtree effect in glucose-limited chemostat cultures of *Saccharomyces cerevisiae*. Appl Environ Microbiol 55:468–477. 2566299 10.1128/aem.55.2.468-477.1989 PMC184133

[75] Lowry OH, Rosebrough NJ, Farr AL, Randall RJ 1951 Protein measurement with the Folin phenol reagent. J Biol Chem 193:265–275. 14907713

[76] Cherry JM, Hong EL, Amundsen C, Balakrishnan R, Binkley G, Chan ET, Christie KR, Costanzo MC, Dwight SS, Engel SR, Fisk DG, Hirschman JE, Hitz BC, Karra K, Krieger CJ, Miyasato SR, Nash RS, Park J, Skrzypek MS, Simison M, Weng S, Wong ED 2012 Saccharomyces Genome Database: the genomics resource of budding yeast. Nucleic Acids Res 40:D700–D705. doi:10.1093/nar/gkr1029. 22110037 PMC3245034

