# Supplementary material for: Requirements for Carnitine Shuttle-Mediated Translocation of Mitochondrial Acetyl Moieties to the Yeast Cytosol
Source: mBio. 2016 May 3;7(3):e00520-16. doi: 10.1128/mBio.00520-16 (PMC4959659; doi:10.1128/mBio.00520-16)
Supplement: Data Set S1 — Original, annotated photographs of spot plates used in Fig. 3, Fig. 4, Fig. 5, and Fig. 6. As spot plate assays were done in duplicate, for each medium, the photos show two plates per strain. Download [file mbo002162799sd1.pdf]

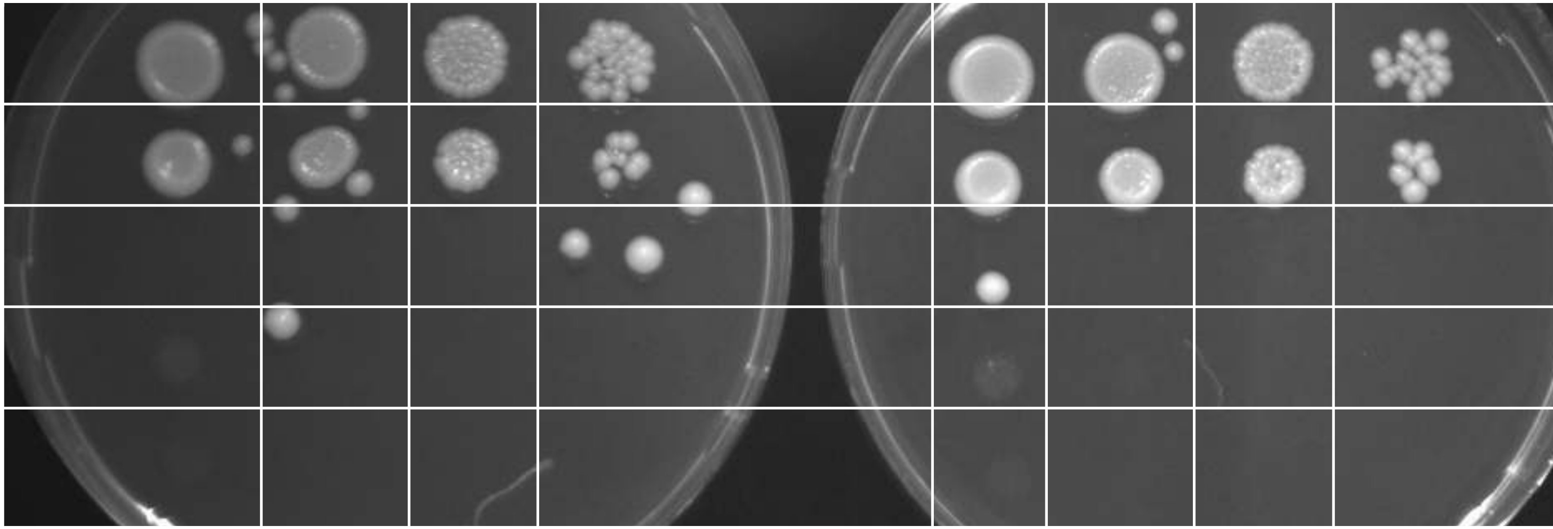

Strains spotted on: 20150218
Photo('s) taken on: 20150222
Medium: SM + 20 g L^{-1} glucose
Used for figure: 3

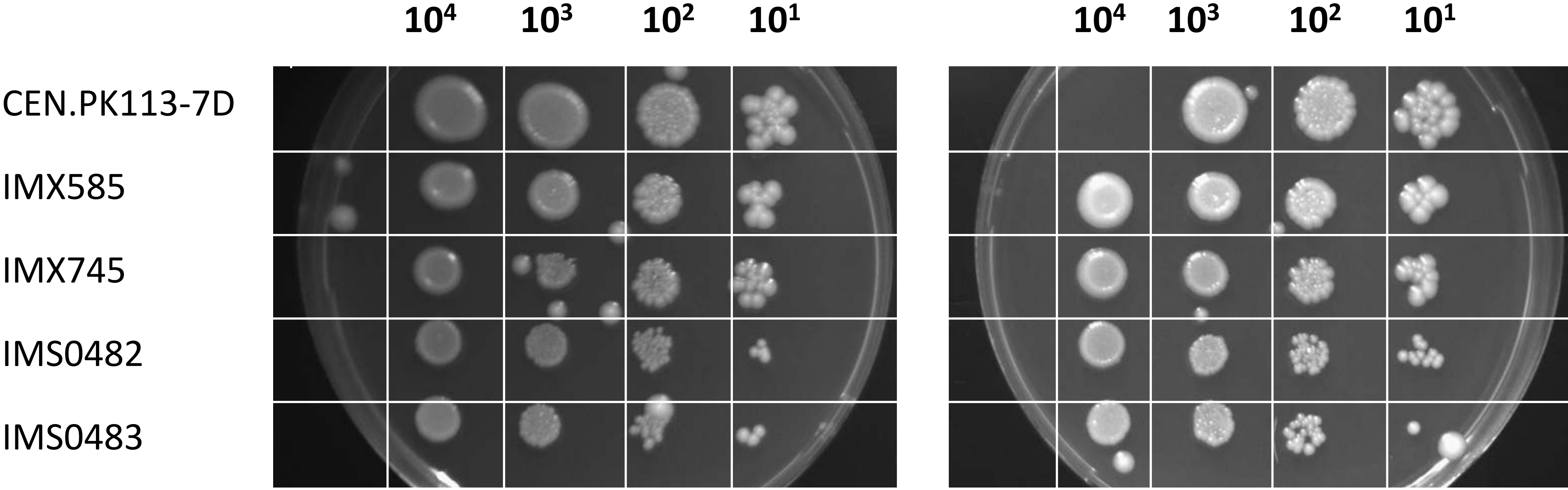

Strains spotted on: 20150218
Photo(‘s) taken on: 20150222
Medium: SM + lipoic acid + 20 g L^{-1} glucose
Used for figure: 3

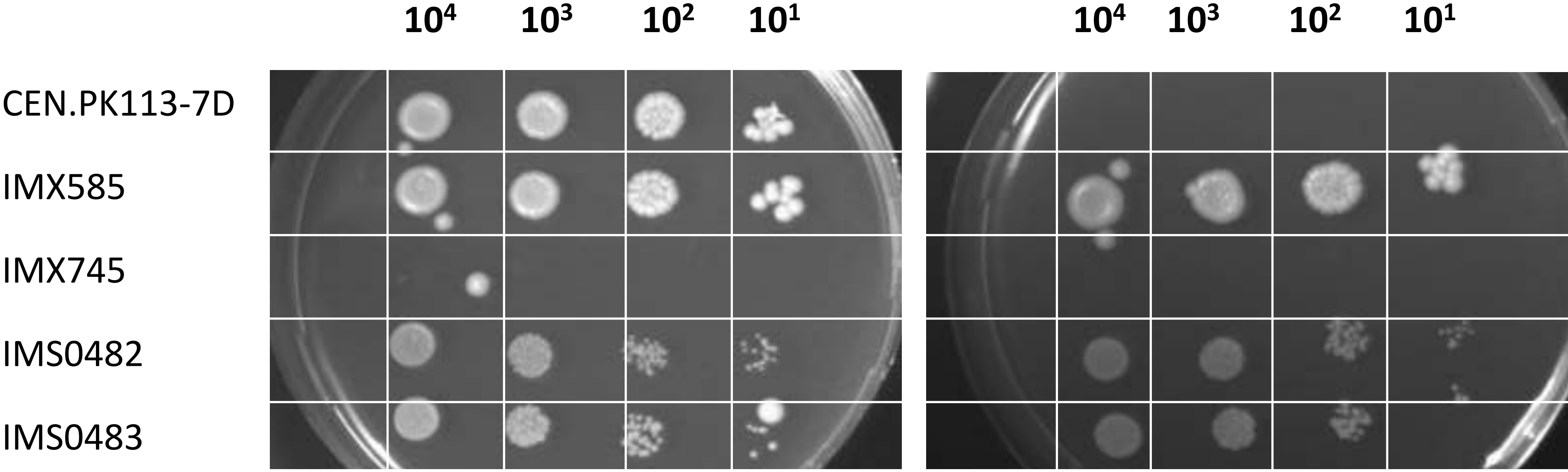

(CEN.PK113-7D was not spotted on this plate)

Strains spotted on: 20150218
Photo('s) taken on: 20150222
Medium: SM + L-carnitine + 20 g L^{-1} glucose
Used for figure: 3

	10^3	10^2	10^1		10^3	10^2	10^1

NDS*
IMW074
IMW075
IMW076
IMW077

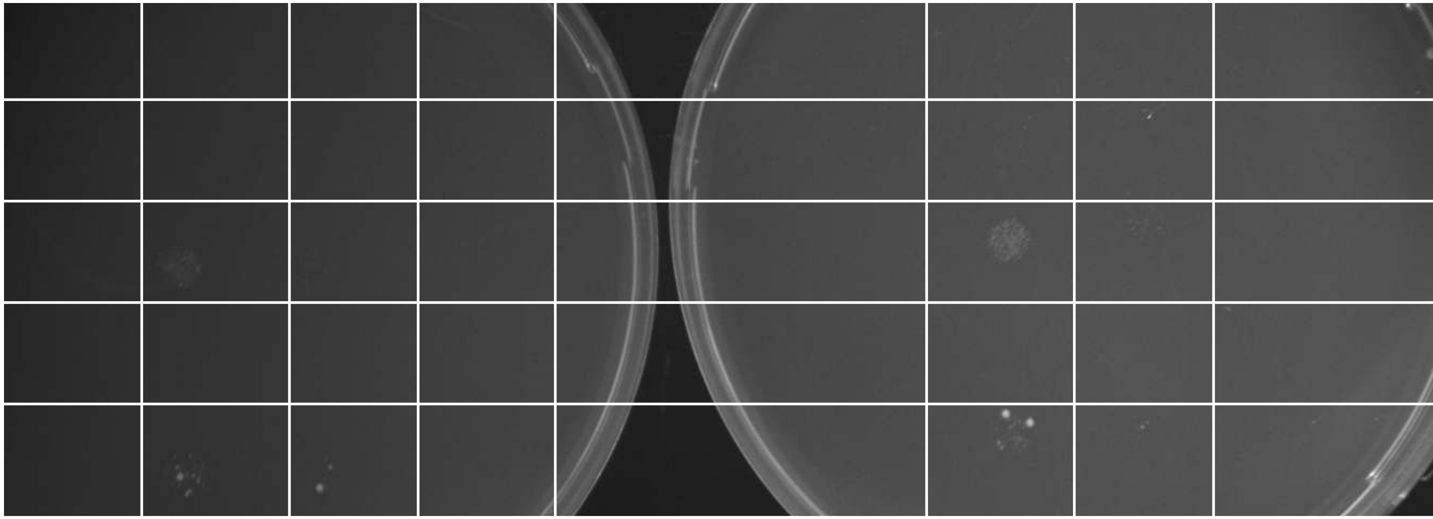

Strains spotted on: 20150417
Photo('s) taken on: 20150421
Medium: SM + 20 g L^{-1} glucose
Used for figure: 4
*NDS, strain not described in this study.

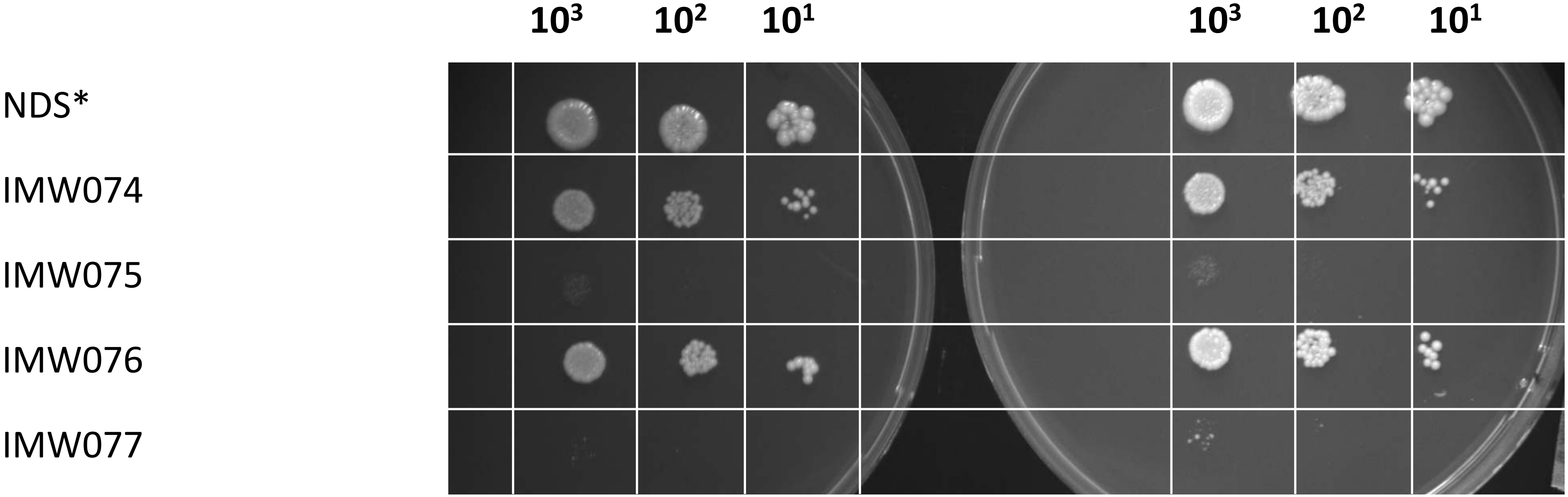

Strains spotted on: 20150417
Photo('s) taken on: 20150421
Medium: SM + lipoic acid + 20 g L^{-1} glucose
Used for figure: 4
*NDS, strain not described in this study.

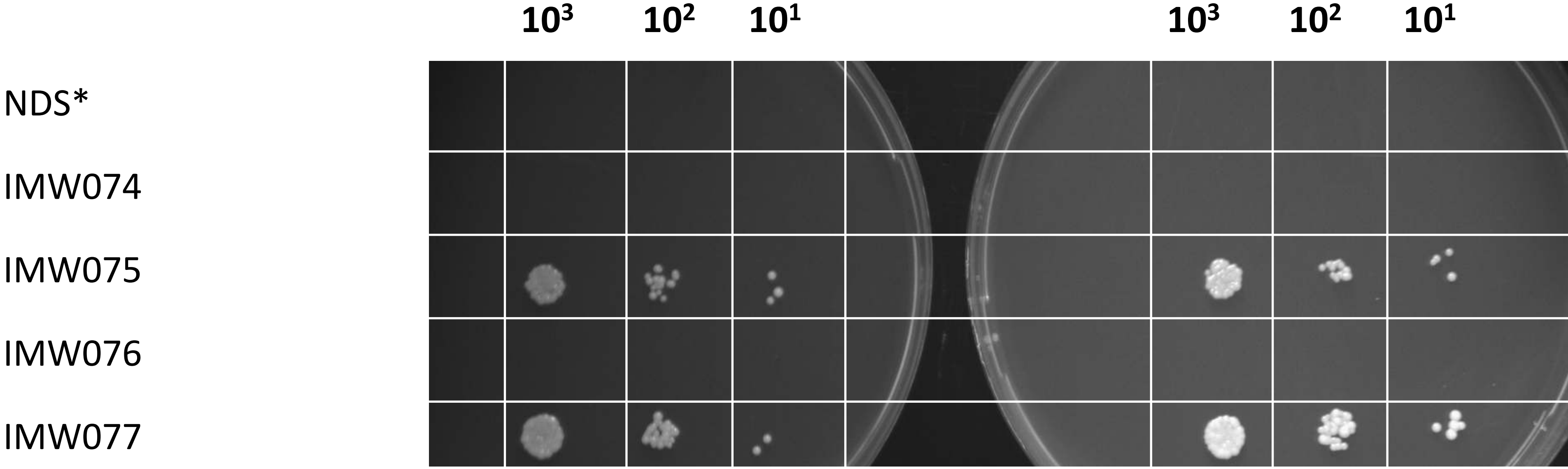

Strains spotted on: 20150417
Photo(‘s) taken on: 20150421
Medium: SM + L-carnitine + 20 g L^{-1} glucose
Used for figure: 4
*NDS, strain not described in this study.

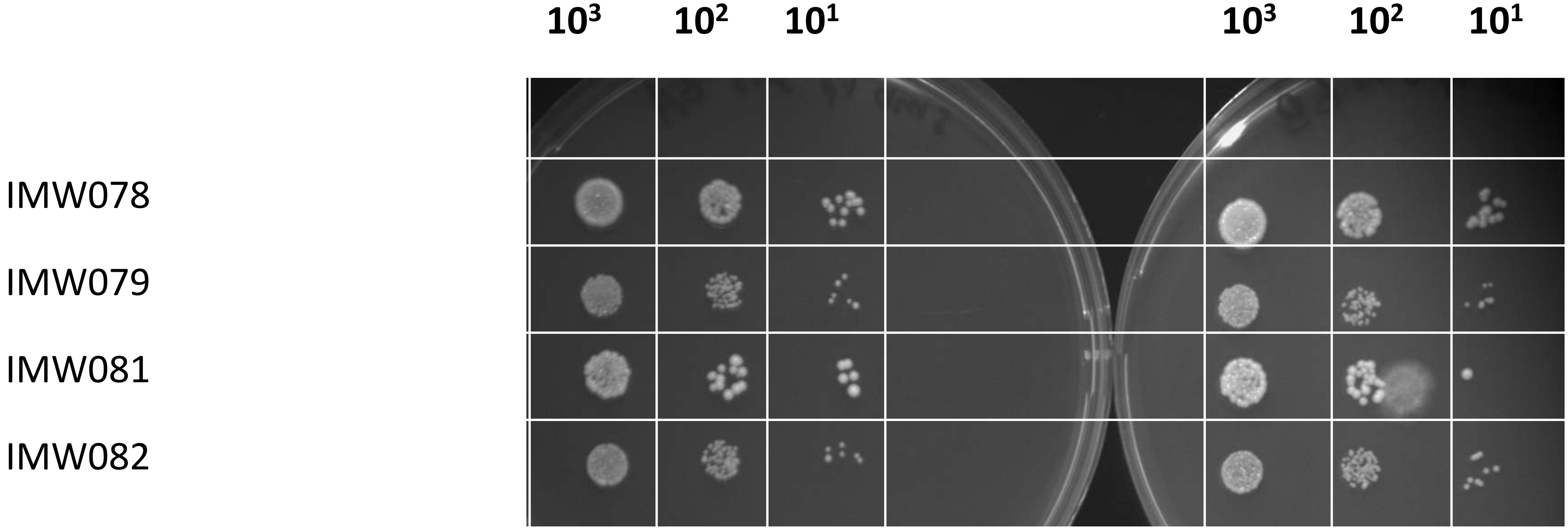

Strains spotted on: 20150612
Photo(‘s) taken on: 20150616
Medium: SM + lipoic acid + 20 g L^{-1} glucose
Used for figure: 5

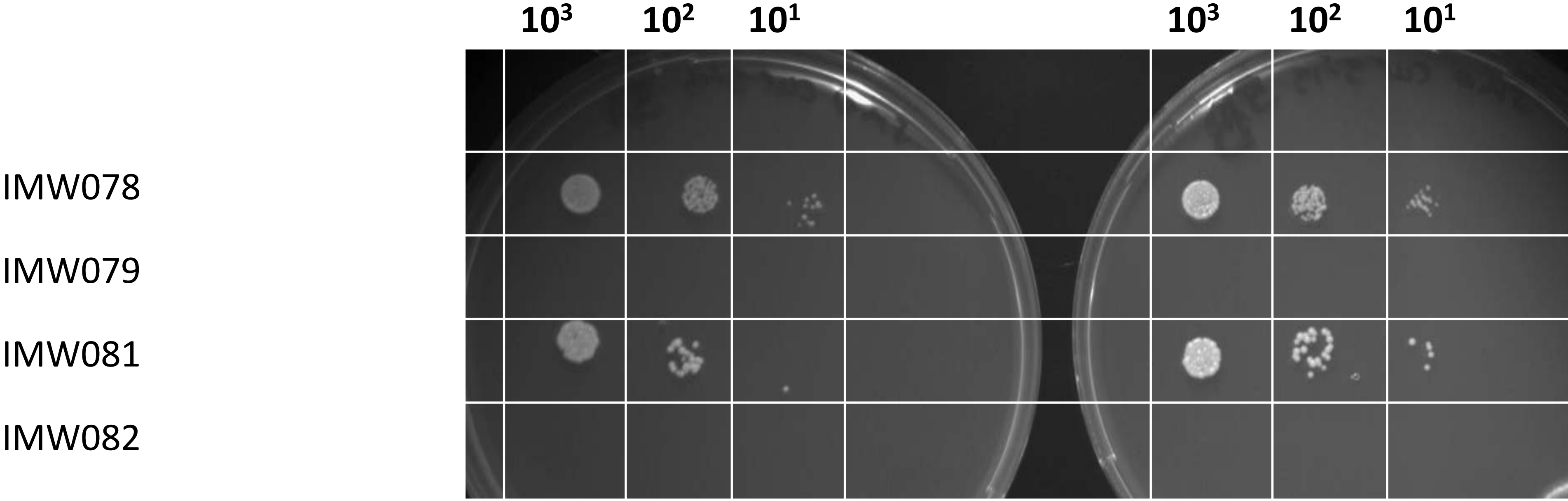

Strains spotted on: 20150612
Photo('s) taken on: 20150616
Medium: SM + L-carnitine + 20 g L^{-1} glucose
Used for figure: 5

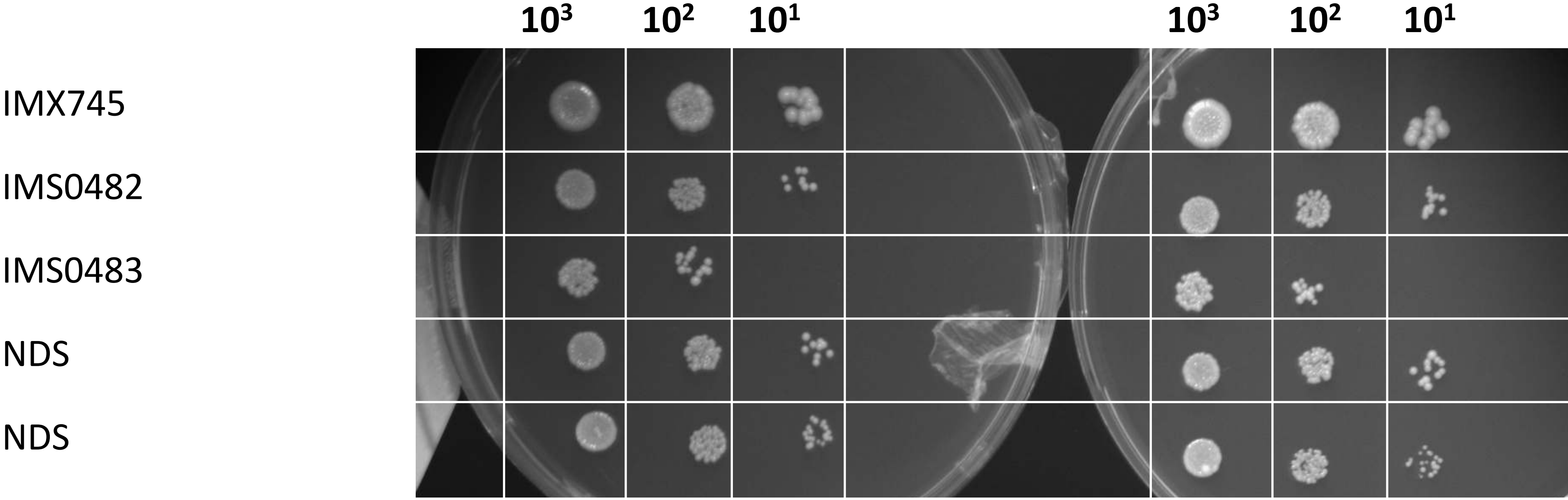

Strains spotted on: 20150526
Photo('s) taken on: 20150530
Medium: SM + lipoic acid + 20 g L^{-1} glucose
Used for figure: 6
*NDS, strain not described in this study.

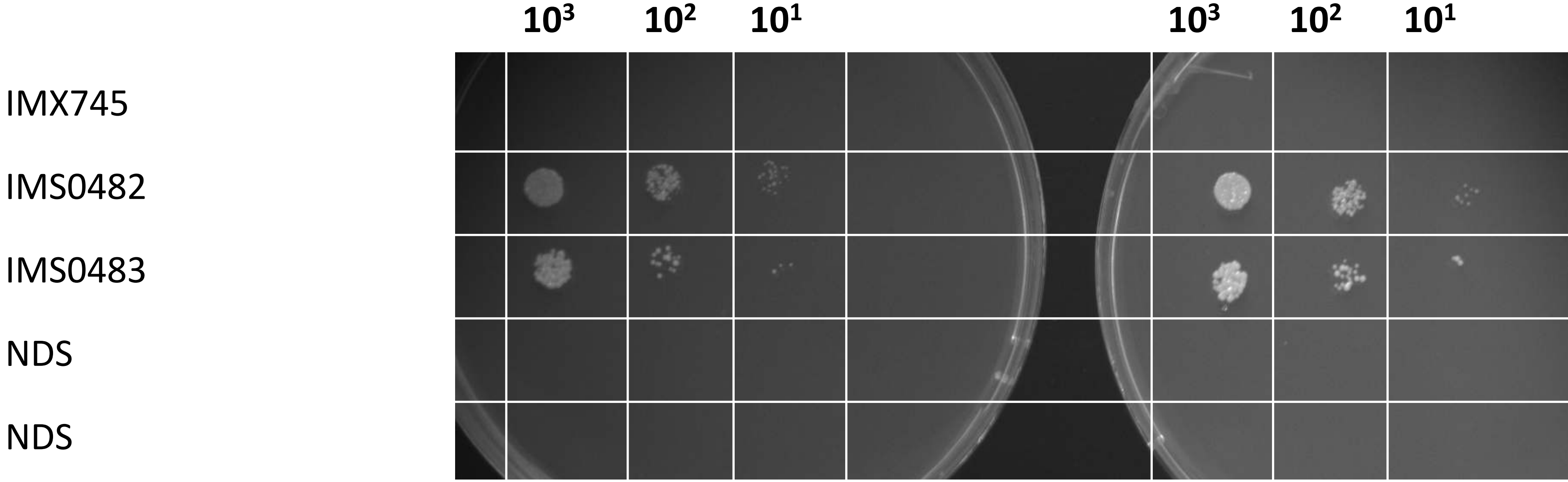

Strains spotted on: 20150526
Photo('s) taken on: 20150530
Medium: SM + L-carnitine + 20 g L^{-1} glucose
Used for figure: 6
*NDS, strain not described in this study.

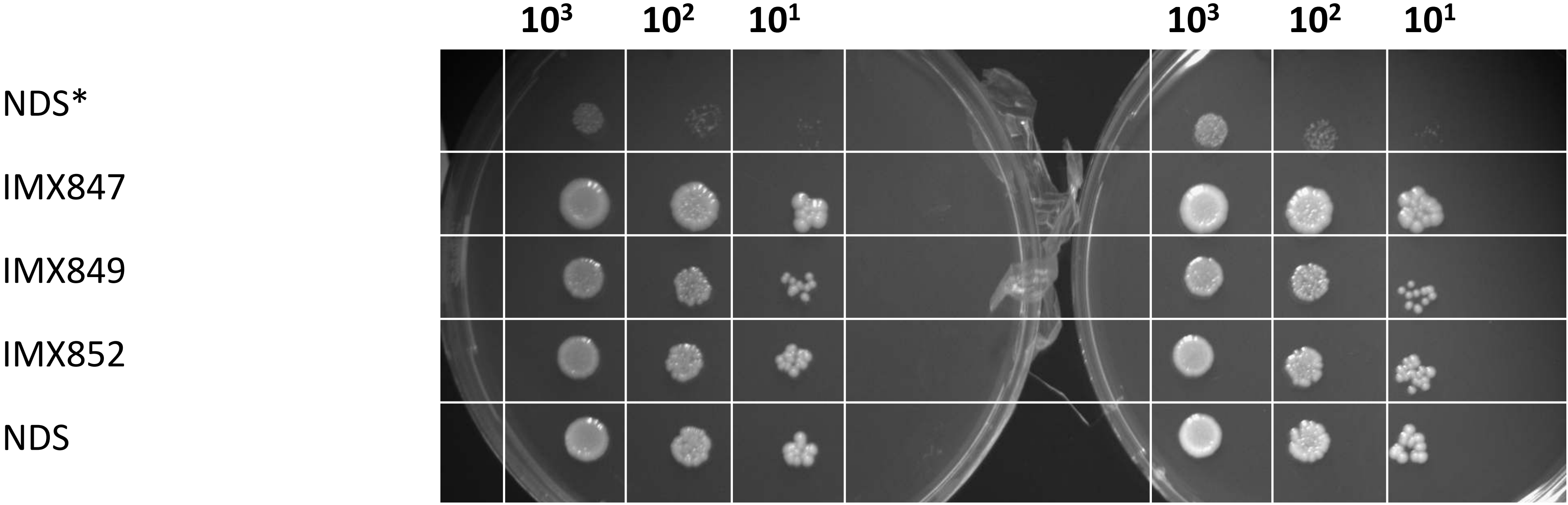

Strains spotted on: 20150526
Photo('s) taken on: 20150530
Medium: SM + lipoic acid + 20 g L^{-1} glucose
Used for figure: 6
*NDS, strain not described in this study.

10^3 10^2 10^1 10^3 10^2 10^1

NDS*

IMX847

IMX849

IMX852

NDS

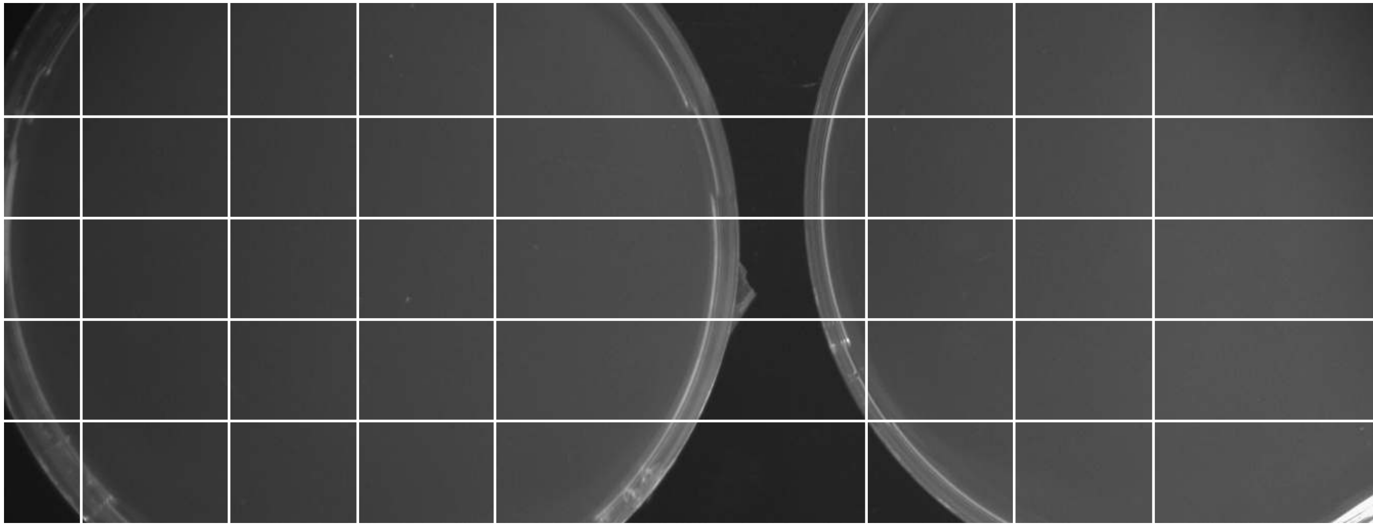

Strains spotted on: 20150526
Photo(‘s) taken on: 20150530
Medium: SM + L-carnitine + 20 g L^{-1} glucose
Used for figure: 6
*NDS, strain not described in this study.

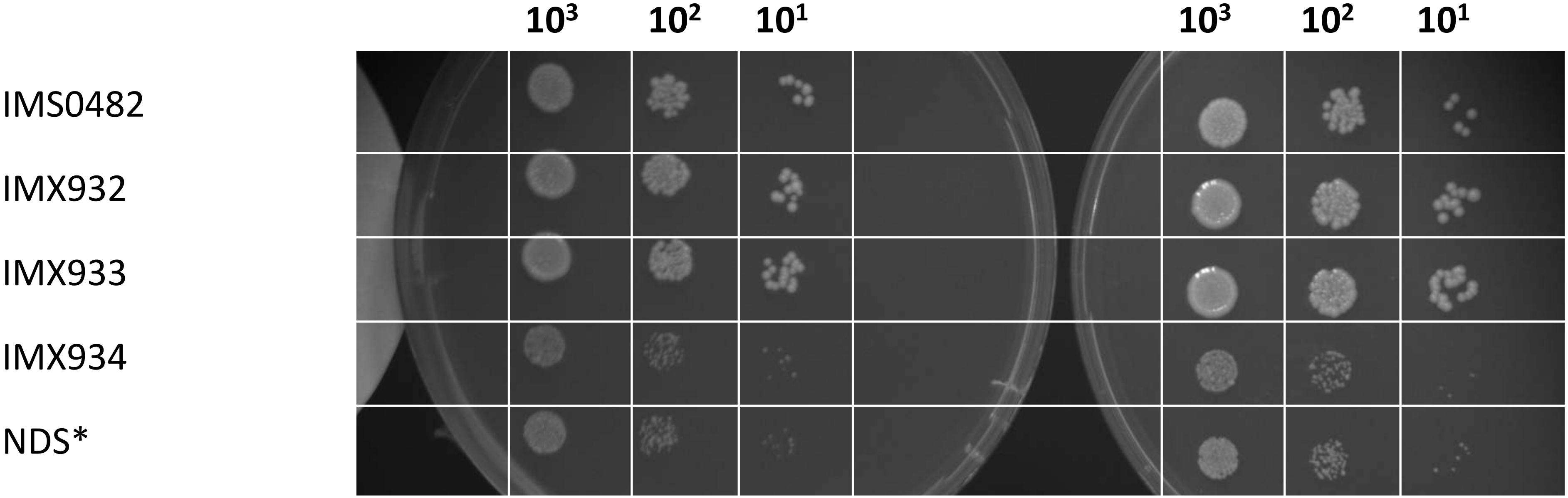

Strains spotted on: 20151027
Photo(‘s) taken on: 20151031
Medium: SM + lipoic acid + 20 g L^{-1} glucose
Used for figure: 6
*NDS, strain not described in this study.

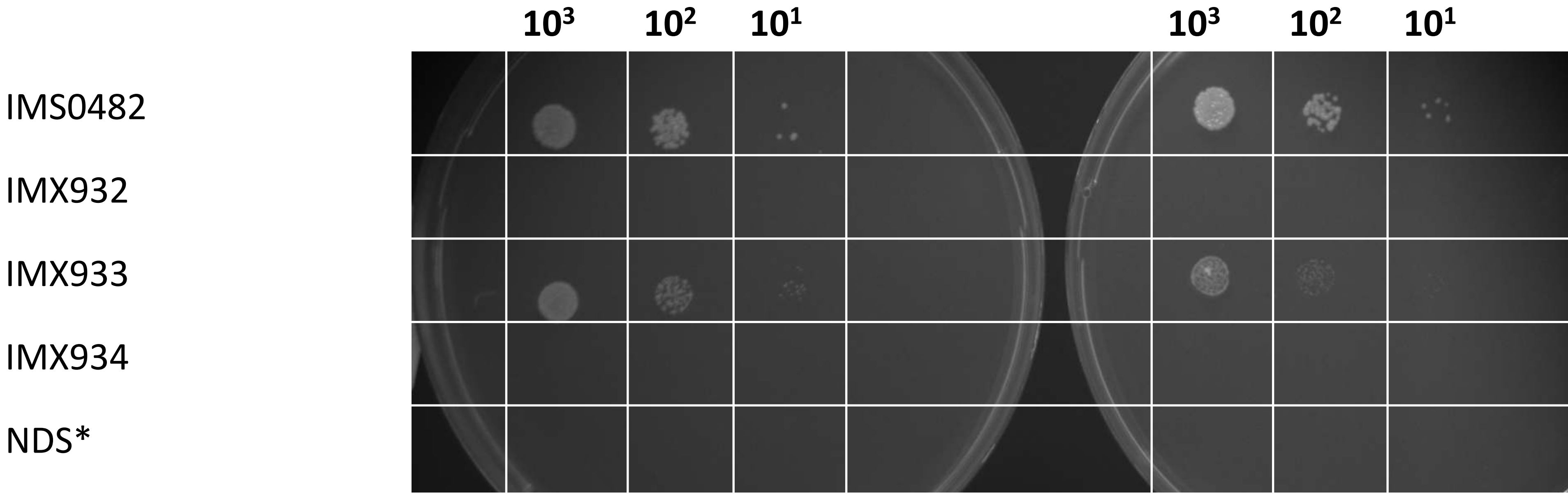

Strains spotted on: 20151027

Photo(‘s) taken on: 20151031

Medium: SM + L-carnitine + 20 g L^{-1} glucose

Used for figure: 6

*NDS, strain not described in this study.

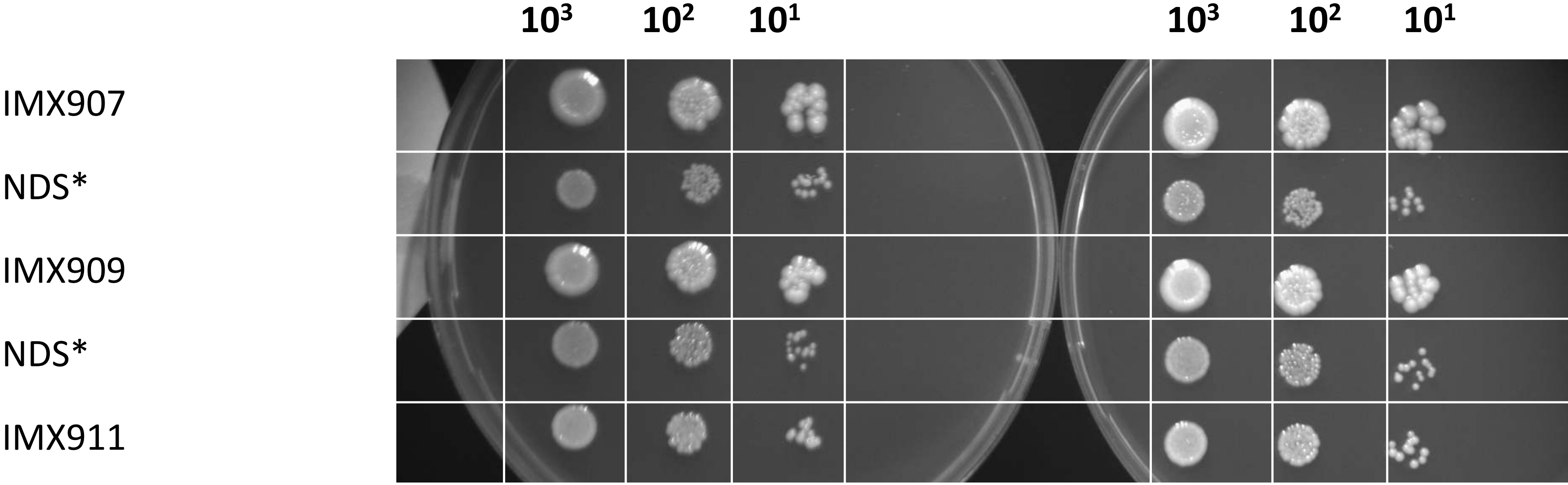

Strains spotted on: 20150717
Photo('s) taken on: 20150721
Medium: SM + lipoic acid + 20 g L^{-1} glucose
Used for figure: 6
*NDS, strain not described in this study.

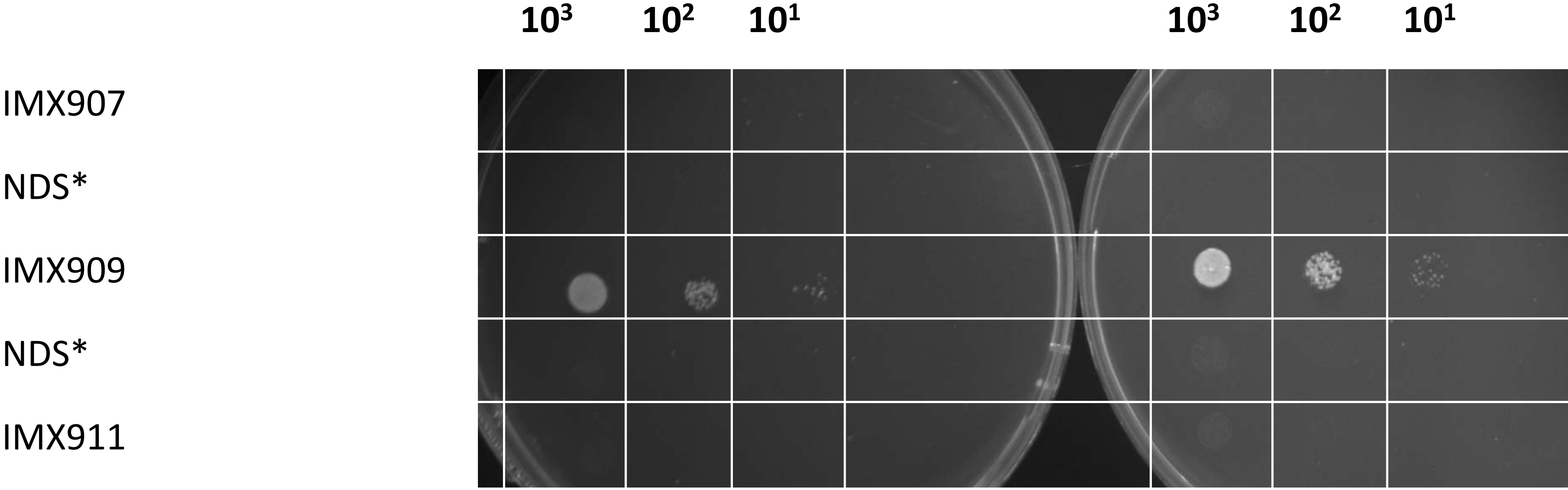

Strains spotted on: 20150717
Photo('s) taken on: 20150721
Medium: SM + L-carnitine + 20 g L^{-1} glucose
Used for figure: 6
*NDS, strain not described in this study.

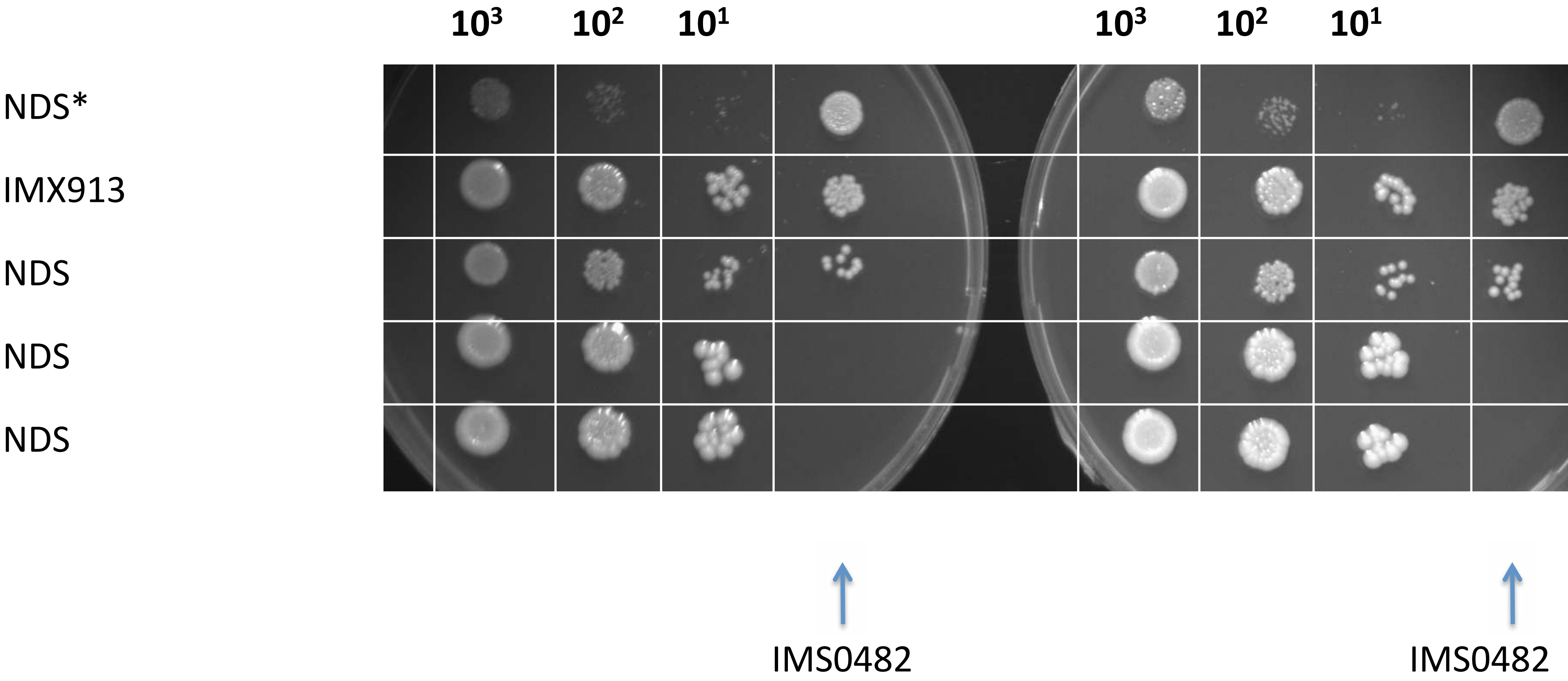

Strains spotted on: 20150717

Photo('s) taken on: 20150721

Medium: SM + lipoic acid + 20 g L^{-1} glucose

Used for figure: 6

*NDS, strain not described in this study.

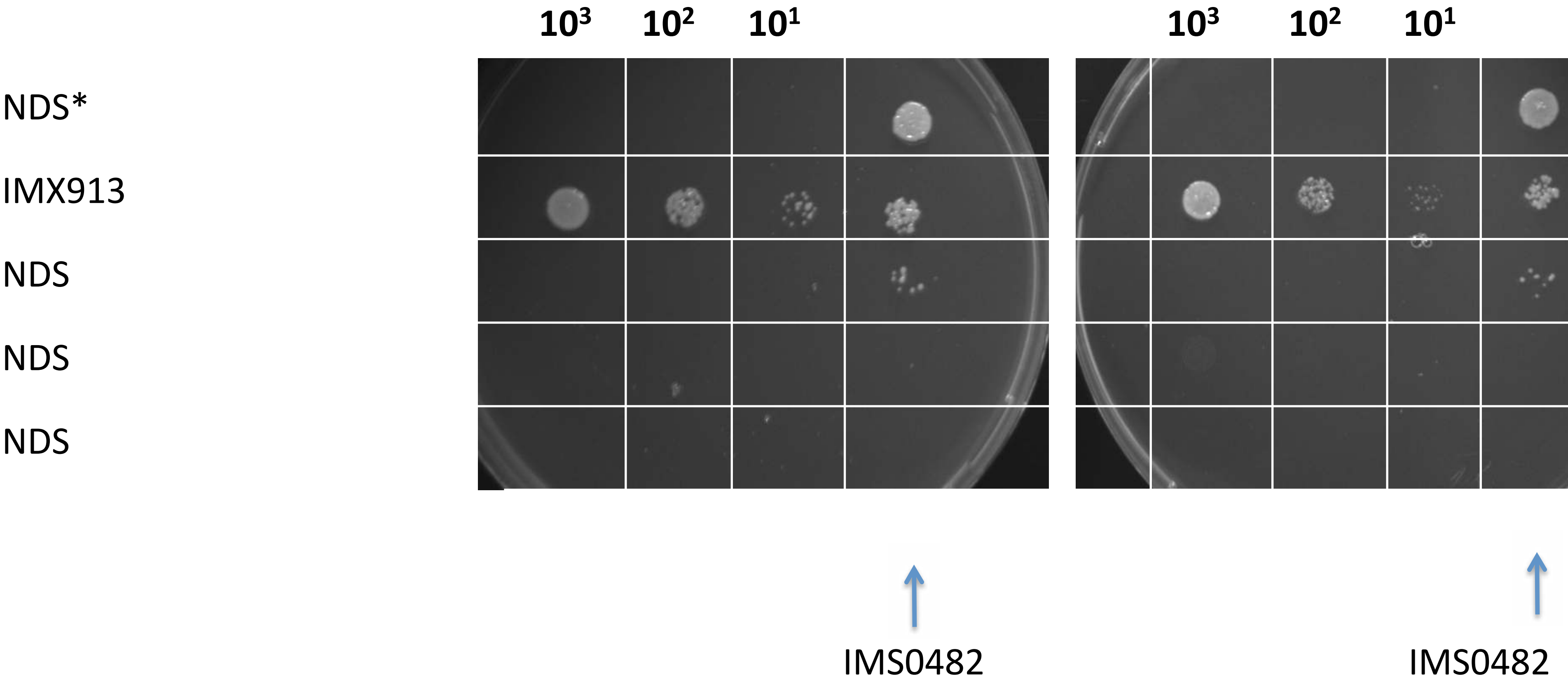

Strains spotted on: 20150717
Photo('s) taken on: 20150721
Medium: SM + L-carnitine + 20 g L^{-1} glucose
Used for figure: 6
*NDS, strain not described in this study.
