# Supplementary material for: Requirements for Carnitine Shuttle-Mediated Translocation of Mitochondrial Acetyl Moieties to the Yeast Cytosol
Source: mBio. 2016 May 3;7(3):e00520-16. doi: 10.1128/mBio.00520-16 (PMC4959659; doi:10.1128/mBio.00520-16)
Supplement: Table S1 — Guide RNA plasmids used in this study. For amplification of the double-guide RNA cassette, a pROS plasmid (33) was used as the template with the primer(s) indicated. [file mbo002162799st1.docx]

| **Table S1. GuideRNA plasmids used in this study.** For amplification of the double guideRNA cassette, a pROS plasmid (33) was used as a template with the primer(s) as indicated. | | | | | |
| --- | --- | --- | --- | --- | --- |
| **Plasmid** | **Target(s)** | **Target sequence(s)** | **Template backbone** | **Relevant primer(s)** | **Comment** |
| pUDR047 | *PDA1* | TACCGGGATCAGACATAGAATGG | pROS12 | 5794 |  |
| pUDR072 | *LplA1* | AGAAACGATTTGTTGATTGACGG | pROS13 | 8016 |  |
| pUDR073 | *pADH1-YAT2* | ATCTCATATACAATGTCAAGCGG | pROS13 | 8014 | Target sequence is at the junction of *ADH1* promoter and *YAT2* ORF |
| pUDR078 | *RTG2* | GCGGTAGTACTCAGTTATCATGG | pROS13 | 8427 |  |
| pUDR079 | *RTG2*, *MCT1* | GCGGTAGTACTCAGTTATCATGG  TAAGAACAGAATTGAACCTAAGG | pROS13 | 8427, 8413 |  |
| pUDR080 | *MCT1* | TAAGAACAGAATTGAACCTAAGG | pROS13 | 8413 |  |
| pUDR085 | *ACH1* | CGAGGCAACGGCCATTAAAGAGG | pROS13 | 6159 |  |
| pUDR105 | Synthetic CRISPRR site | TGTAGAATTTCACCTAGACGTGG | pROS12 | 8558 |  |
| pUDR119 | *SGA1* | ATTGACCACTGGAATTCTTCCGG | pROS11 |  | Constructed earlier, see (8) |
