# Supplementary material for: Requirements for Carnitine Shuttle-Mediated Translocation of Mitochondrial Acetyl Moieties to the Yeast Cytosol
Source: mBio. 2016 May 3;7(3):e00520-16. doi: 10.1128/mBio.00520-16 (PMC4959659; doi:10.1128/mBio.00520-16)
Supplement: Table S2 — Primers used in this study. [file mbo002162799st2.docx]

| **Table S2. Primers used in this study** | | |
| --- | --- | --- |
| **Number** | **Name** | **Sequence 5’ → 3’** |
| **Primers for guideRNA cassette construction** | | |
| 6005 | p426 CRISP rv | GATCATTTATCTTTCACTGCGGAGAAG |
| 5794 | pCAS9 target pda1 fw | TGCGCATGTTTCGGCGTTCGAAACTTCTCCGCAGTGAAAGATAAATGATCTACCGGGATCAGACATAGAAGTTTTAGAGCTAGAAATAGCAAGTTAAAATAAGGCTAGTCCGTTATCAAC |
| 6159 | ACH1 gRNA | GTGCGCATGTTTCGGCGTTCGAAACTTCTCCGCAGTGAAAGATAAATGATCCGAGGCAACGGCCATTAAAGGTTTTAGAGCTAGAAATAGCAAGTTAAAATAAG |
| 8016 | LplA1 target_RNA FW | TGCGCATGTTTCGGCGTTCGAAACTTCTCCGCAGTGAAAGATAAATGATCAGAAACGATTTGTTGATTGAGTTTTAGAGCTAGAAATAGCAAGTTAAAATAAG |
| 8014 | pADH1-YAT2 target_RNA FW | TGCGCATGTTTCGGCGTTCGAAACTTCTCCGCAGTGAAAGATAAATGATCATCTCATATACAATGTCAAGGTTTTAGAGCTAGAAATAGCAAGTTAAAATAAG |
| 8427 | RTG2_targetRNA FW RsaI | TGCGCATGTTTCGGCGTTCGAAACTTCTCCGCAGTGAAAGATAAATGATCGCGGTAGTACTCAGTTATCAGTTTTAGAGCTAGAAATAGCAAGTTAAAATAAG |
| 8413 | MCT1_targetRNA-L214W FW EcoRI | TGCGCATGTTTCGGCGTTCGAAACTTCTCCGCAGTGAAAGATAAATGATCTAAGAACAGAATTGAACCTAGTTTTAGAGCTAGAAATAGCAAGTTAAAATAAG |
| 8558 | CRISPRR target gRNA | TGCGCATGTTTCGGCGTTCGAAACTTCTCCGCAGTGAAAGATAAATGATCTGTAGAATTTCACCTAGACGGTTTTAGAGCTAGAAATAGCAAGTTAAAATAAG |
| **CRISPR *acs2Δ* locus** | | |
| 7349 | ACS2_repair oligo rv | AAAATAGAAAACAGAAAAGGAGCGAAATTTTATCTCATTACGAAATTTTTCTCATTTAAGATTTTATTATTGTATTGATTTACTTTCCTGTATTCTGTTTGTGTATAACAATCACTAACC |
| 7350 | ACS2_repair oligo fw | GGTTAGTGATTGTTATACACAAACAGAATACAGGAAAGTAAATCAATACAATAATAAAATCTTAAATGAGAAAAATTTCGTAATGAGATAAAATTTCGCTCCTTTTCTGTTTTCTATTTT |
| **CRISPR *sga1Δ* locus** | |  |
| 8012 | SGA1_repair oligo fw | ATTTACAATATAGTGATAATCGTGGACTAGAGCAAGATTTCAAATAAGTAACAGCAGCAAACAAAAAAAAATAAAAGAAAAGCGAGAAGTATACACAAGTGTATTTCCTAGATATTTACA |
| 8013 | SGA1_repair oligo rv | TGTAAATATCTAGGAAATACACTTGTGTATACTTCTCGCTTTTCTTTTATTTTTTTTTGTTTGCTGCTGTTACTTATTTGAAATCTTGCTCTAGTCCACGATTATCACTATATTGTAAAT |
| **CRISPR *MCT1^T641G^* mutation and *MCT1* deletion** | | |
| 8417 | MCT1_repair oligo L214W fw | GTAAAGCAATGTGTAGTCACCGGTCTGGTTGATGATTTAGAGTCCTTAAGAACAGAATGGAACCTAAGGTTCCCGCGTTTAAGAATTACAGAATTAACTAACCCATACAACATCCCCTTC |
| 8418 | MCT1_repair oligo L214W rv | GAAGGGGATGTTGTATGGGTTAGTTAATTCTGTAATTCTTAAACGCGGGAACCTTAGGTTCCATTCTGTTCTTAAGGACTCTAAATCATCAACCAGACCGGTGACTACACATTGCTTTAC |
| 8415 | MCT1_repair oligo fw | AGGGTAGTAACAAAGCGTTTTGCACTTTTCTGATCGTGGTACACATATATAAGCGTTTGTGAAAGAGATCAAAACTGCGAACTCTCTCCACTCCCAGTCTGTGCTTCTACGCATTCATTG |
| 8416 | MCT1_repair oligo rv | CAATGAATGCGTAGAAGCACAGACTGGGAGTGGAGAGAGTTCGCAGTTTTGATCTCTTTCACAAACGCTTATATATGTGTACCACGATCAGAAAAGTGCAAAACGCTTTGTTACTACCCT |
| **CRISPR *RTG2^G503T^* mutation and *RTG2* deletion** | | |
| 8430 | RTG2_repair oligo W168L fw | CATTTAATACAGTAAGAGGTCTATATCTAGATGTGGCAGGCGGTAGTACTCAGTTATCATTGGTAATAAGCTCGCACGGAGAAGTCAAGCAATCCAGCAAACCTGTATCTTTGCCATATG |
| 8431 | RTG2_repair oligo W168L rv | CATATGGCAAAGATACAGGTTTGCTGGATTGCTTGACTTCTCCGTGCGAGCTTATTACCAATGATAACTGAGTACTACCGCCTGCCACATCTAGATATAGACCTCTTACTGTATTAAATG |
| 8428 | RTG2_repair oligo fw | ACTCTTGGAAGTGTCCTTTACTAAGGATTGTTTTGAACGAAAAGTGTAGGCGTGCCACAAAGACATCTAGTCTTTAAATACTTGAACAATAAATACGAAATCCTTATATAAGCATCTTTT |
| 8429 | RTG2_repair oligo rv | AAAAGATGCTTATATAAGGATTTCGTATTTATTGTTCAAGTATTTAAAGACTAGATGTCTTTGTGGCACGCCTACACTTTTCGTTCAAAACAATCCTTAGTAAAGGACACTTCCAAGAGT |
| **CRISPR *pADH1-yat2(-11,174)::CRISPRR* site incorporation and *YAT2* deletion** | | |
| 8621 | YAT2 repair oligo CRISPRR fw | GTTTCTTTTTCTGCACAATATTTCAAGCTATACCAAGCATACAATCAACTAGTAGAATTTCACCTAGACGTGGGAACAAATTGAGAAGCTGTCATCCATAATAAGAGATTTTGAAGAG |
| 8622 | YAT2 repair oligo CRISPRR rv | CTCTTCAAAATCTCTTATTATGGATGACAGCTTCTCAATTTGTTCCCACGTCTAGGTGAAATTCTACTAGTTGATTGTATGCTTGGTATAGCTTGAAATATTGTGCAGAAAAAGAAAC |
| 8874 | pADH1-YAT2 repair oligo fw | TTTCTTCCTTGTTTCTTTTTCTGCACAATATTTCAAGCTATACCAAGCATACAATCAACTGAACGCTCTTTGTTTATCTATTTATTACTAGCATTATGCGTAAGCTTGGCGTGATGTGAT |
| 8875 | pADH1-YAT2 repair oligo rv | ATCACATCACGCCAAGCTTACGCATAATGCTAGTAATAAATAGATAAACAAAGAGCGTTCAGTTGATTGTATGCTTGGTATAGCTTGAAATATTGTGCAGAAAAAGAAACAAGGAAGAAA |
| **CRISPR *PDA1* deletion** | | |
| 6157 | PDA1 repair fw | TGTTTATCTCTCTTCTGATTCCTCCACCCCTTCCTTACTCAACCGGGTAAATGTCGCATCTTAATCGTAAGGAAAAATAAAATAATAGTGCTGTGATCGCATGATATTCTTCCCTGGAAG |
| 6158 | PDA1 repair rv | CTTCCAGGGAAGAATATCATGCGATCACAGCACTATTATTTTATTTTTCCTTACGATTAAGATGCGACATTTACCCGGTTGAGTAAGGAAGGGGTGGAGGAATCAGAAGAGAGATAAACA |
| **CRISPR *ACH1* deletion** | | |
| 6160 | ACH1 repair fw | GACAATAGCGGCAAAACAAACAACACATTTCTTTTTTTCTTTTTCACATATTGCACTAAATGTTTGTGCGCAAACCGAGAGATGAGTATTTAAAAAAAAAAGAAAGGAAATGATATGATT |
| 6161 | ACH1 repair rv | AATCATATCATTTCCTTTCTTTTTTTTTTAAATACTCATCTCTCGGTTTGCGCACAAACATTTAGTGCAATATGTGAAAAAGAAAAAAAGAAATGTGTTGTTTGTTTTGCCGCTATTGTC |
| **Marker cassette *CAT2* deletion** | | |
| 9237 | CAT2-S1 | ATGAGGATCTGTCATTCGAGAACTCTCTCAAACTTAAAGGCAGCTGAAGCTTCGTACGC |
| 9238 | CAT2-S2 | ATTGATATGCAGCCACTCGTTATTCAACATGTATGCCAGTGCATAGGCCACTAGTGGATCTG |
| **Marker cassette *YAT1* deletion** | | |
| 9239 | YAT1-S1 | ATGCCAAACTTAAAGAGACTACCCATCCCGCCACTGCAGGCAGCTGAAGCTTCGTACGC |
| 9240 | YAT1-S2 | GTAATCGTAGCCGGACAACAGGTATTTCATGTCTTCTGATGCATAGGCCACTAGTGGATCTG |
| **Primers for amplification of the *YAT2^C173G^* mutation** | | |
| 8618 | pADH1 fw | CCTCGTCATTGTTCTCGTTC |
| 8619 | YAT2 rv | GTTTAGATTGCAGCTTCTCAC |
| **Primers for amplification of the *pADH1-YAT2-tYAT2* cassettes** | | |
| 8647 | tag C fw ol pSGA1 | TTTACAATATAGTGATAATCGTGGACTAGAGCAAGATTTCAAATAAGTAACAGCAGCAAAACGTCTCACGGATCGTATATGC |
| 8648 | tag J rv (ol tSGA1) | TATATTTGATGTAAATATCTAGGAAATACACTTGTGTATACTTCTCGCTTTTCTTTTATTCGACGAGATGCTCAGACTATG |
| 8902 | tag J fw (ol pRS426) | CTATAGGGCGAATTGGGTACCGGGCCCCCCCCGACGAGATGCTCAGACTATG |
| 8903 | tag C rv (ol pRS426) | GAACTAGTGGATCCCCCGGGCTGCAGGAATTACGTCTCACGGATCGTATATG |
| **Primers for amplification of *CAT2* ORF and pRS426-pTDH3…tCYC1 plasmid** | | |
| 3627 | pTDH3 rv | TTTGTTTGTTTATGTGTGTTTATTCGAAAC |
| 3921 | tCYC1 fw (smaller) | CAGGCCCCTTTTCCTTTG |
| 5948 | pRS426-CAT2 fw | TTTAAAACACCAAGAACTTAGTTTCGAATAAACACACATAAACAAACAAAATGAGGATCTGTCATTCGAGAAC |
| 5949 | pRS426-CAT2 rv | TAAGCGTGACATAACTAATTACATGATATCGACAAAGGAAAAGGGGCCTGTCAGTGATGGTGGTGATGATGTAACTTTGCTTTTCGTTTATTCTCATTTTC |
| **Confirmation of *PDA1* deletion** | | |
| 3146 | ROSP031 DG PDA1 KO fw | ATCGCGCGTGTACATGTC |
| 3147 | ROSP032 DG PDA1 KO rv | GCGGCTATTTTCCGGTCTG |
| **Confirmation of *ACH1* deletion** | | |
| 3770 | ACH1kocheck2f | CGGGCTTACATTAGCACAC |
| 3771 | ACH1kocheck2r | GCAAGAAAAAACAACGCATTGG |
| **Confirmation of *sga1Δ* locus** | | |
| 4223 | SGA1 outside fw | CTTGGCTCTGGATCCGTTATCTG |
| 4229 | Sequence SGA1 2 rv | TGGTCGACAGATACAATCCTGG |
| **Confirmation of *acs2Δ* locus** | | |
| 2618 | acs2 Ctrl Fw | TACCCTATCCCGGGCGAAGAAC |
| 2619 | acs2 KO Ctrl Rv | CCGATATTCGGTAGCCGATTCC |
| **Confirmation of *cat2Δ* and *yat1Δ* loci** | | |
| 9091 | K1-A | GGATGTATGGGCTAAATGTACG |
| 9092 | K2 | GTTTCATTTGATGCTCGATGAG |
| 9265 | CAT2-A1 | TTCACCTCTTCTCAACGCTG |
| 9266 | CAT2-A4 | CAGGCGTACCACGAGATAG |
| 9267 | YAT1-A1 | ACGATTACATACAAGATGAACG |
| 9268 | YAT1-A4 | TTCGTTTAGATCAAGATGGATG |
| **Confirmation of SNPs** | | |
| 8423 | MCT1_dg fw-2 | GAGTCAGAACGGCAAGGAATC |
| 8426 | MCT1_dg rv-3 | CTCCATCAACGTGTGAGTTC |
| 8507 | RTG1 inside DG fw | ACAGAAGCCACGCGAGATG |
| 8508 | RTG1 inside DG rv | AGAAGCAGATGTCCCATACC |
| 706 | 5Padh1-3 | gtcgttgttccagagctgatgag |
| 7497 | YAT2_3 | AAAGCGTCATCTGCGAGAACC |
