# Supplementary material for: Requirements for Carnitine Shuttle-Mediated Translocation of Mitochondrial Acetyl Moieties to the Yeast Cytosol
Source: mBio. 2016 May 3;7(3):e00520-16. doi: 10.1128/mBio.00520-16 (PMC4959659; doi:10.1128/mBio.00520-16)
Supplement: Table S3 — Sequences of the YAT2 and CAT2 gene cassettes used in this study. The sequences of the open reading frames are underlined, and the C173G mutation is indicated in red. [file mbo002162799st3.docx]

| **Table S3. Sequences of the *YAT2* and *CAT2* gene cassettes used in this study**. The sequences of the open reading frames are underlined and the C173G mutation is indicated in red. | |
| --- | --- |
| *pADH1-YAT2-tYAT2* | CTATAGGGCGAATTGGGTACCGGGCCCCCCCCGACGAGATGCTCAGACTATGTGTTCTACCTGCTTGGACATCTTCGCGTATATGACGGCCCCCGGGAAGGTGAGACGCGCATAACCGCTAGAGTACTTTGAAGAGGAAACAGCAATAGGGTTGCTACCAGTATAAATAGACAGGTACATACAACACTGGAAATGGTTGTCTGTTTGAGTACGCTTTCAATTCATTTGGGTGTGCACTTTATTATGTTACAATATGGAAGGGAACTTTACACTTCTCCTATGCACATATATTAATTAAAGTCCAATGCTAGTAGAGAAGGGGGGTAACACCCCTCCGCGCTCTTTTCCGATTTTTTTCTAAACCGTGGAATATTTCGGATATCCTTTTGTTGTTTCCGGGTGTACAATATGGACTTCCTCTTTTCTGGCAACCAAACCCATACATCGGGATTCCTATAATACCTTCGTTGGTCTCCCTAACATGTAGGTGGCGGAGGGGAGATATACAATAGAACAGATACCAGACAAGACATAATGGGCTAAACAAGACTACACCAATTACACTGCCTCATTGATGGTGGTACATAACGAACTAATACTGTAGCCCTAGACTTGATAGCCATCATCATATCGAAGTTTCACTACCCTTTTTCCATTTGCCATCTATTGAAGTAATAATAGGCGCATGCAACTTCTTTTCTTTTTTTTTCTTTTCTCTCTCCCCCGTTGTTGTCTCACCATATCCGCAATGACAAAAAAATGATGGAAGACACTAAAGGAAAAAATTAACGACAAAGACAGCACCAACAGATGTCGTTGTTCCAGAGCTGATGAGGGGTATCTCGAAGCACACGAAACTTTTTCCTTCCTTCATTCACGCACACTACTCTCTAATGAGCAACGGTATACGGCCTTCCTTCCAGTTACTTGAATTTGAAATAAAAAAAAGTTTGCTGTCTTGCTATCAAGTATAAATAGACCTGCAATTATTAATCTTTTGTTTCCTCGTCATTGTTCTCGTTCCCTTTCTTCCTTGTTTCTTTTTCTGCACAATATTTCAAGCTATACCAAGCATACAATCAACTATCTCATATACAATGTCAAGCGGCAGTACTATTGTTTCGTCTGACAAGTCGGAGCGAACATTTAAGCATGAGGAAGAACTACCCAAGTTACCATTACCTAAACTCCGTGACACTTTGCAGCGTCTGAAGGAGAGCTTAGAGCCATTGTATTATGCTGATGGTTACTATCAACATCCTTTAGATCCAGAACAAATTGAGAAGCTGTCATCCATAATAAGAGATTTTGAAGAGAATCCCGTAAGTGAGAAGCTGCAATCTAAACTACAGAGCTACCATGACACTAGGGATTGTTACCTTGATGAATTACACTTAGACATCAATAACCAGACCTCTACTAGGGAGATTCAGGACGATGTCCTACCCAGGAATCCCTTCTTGGTTCTCGCAGATGACGCTTTGCCAAACATCACTCAAGCTGATAGGTCAGCCGTCCTAGTGCACTCTGCGGCCAGGTTTATTTCTGCTTTGAAACAAGATTTGTTGCCACCAGATATCAATGCCACCAATGGCAAACCACTGTCAATGGCTCCATTTCTGAACTTGTTCGGTACTACTCGTAGTCCTGTTTTTCAGCGCGGTGAGGTTGAAAATTTTGATTTGAACAAACCTTACACAGCATCTGATTTAGAAGACCCTGATTATTCAAGCGATGAGGGCGATAACGACGAACCCACCCAAAAAGACTTTGATGACCGTAAAAGAAAACACGAAGAAGACATATTCACTGGCAATGGTATAACTATAAAAAGACATCCGGATAGCAAACACATCTTGATCATCTCCAGGGGCCAATACTATACACTGGAAGTACTTGATTCGACGAATAAAATTATATACACTGCTGCAGAATTAACCACAATTTTCAACCATATAATAAAAGATTCTTCTGGCATTGAAAAATCAACTGCGTTAGGTAGTTTGACCTCTCACTCTTTTAGAAACTGGAAATATGCAAGGAAGAGACTACAGAAAAGGTATCCTAATGAATTACATCGCATAGACTCTGCTCTGTTCGTGTTAGTGCTGGACGAGTCACAAGAAGAAACCACCAATGATGGTGATGATACCGCTGATATCAGTCAAATGTTTAACAGGACCATCACTGAGCGAGACAAGAAATGTACTTCCGCCAATTGTAAGAGAGTGTTTTACGGTACTTCTATAATAAATAGCAAAGGCCATCAAGTGGGTTCATGTGTTTCTCGTTGGTATGATAAATTACAGTTAGTTGTCACTGCAGATGCAAAAGCCACAGTGATTTGGGATTCCTTCACATGTGACGGGTCAGTCGTGCTTCGGTTTACTTCTGAAATTTATACAGAATCAGTCTTGAGATTAGCTAGAGACGTTAACGCCGGTGACCCACAGTTTTCACTCTGGCCTAACGTGACACAAATGGATCCTGAAACTAAAAAACTGATGACGGCGACCATAAGTGCCGACGGCGGAGGCCCTTCTGAAATTGACCCTAAGTTGGTTGTAAATAAAATCGATTGGTCTTTCAGTAACATCCTGAACACACATGTCCATTTGTCAGAAACCAAACTAGCTGATCTGATTTCCAAGTATGATATTGTTCGCGCTTCTATTCCATTGGGCAGAAGGTCAGCTCAAAGATTAGGAGTCAAGCCCGATTCGATGGTTCAAGTAGCTTTACAAATAGCTCACTATGCTTTGTATGGAAGAATGGTGTTTGGATTAGAACCAGTATCCACACGTGGTTTCAAAAACTCGAGATCATCTTTTATTAACATTCAAAGTCAAGCGCTGTTGGAACTATGTCAATTGTTTATTTCTAGCTCCATTGATGGGACAGATAAATTAGACAAATTTATCCAAACCTGCGAAACACATAATAACATGGTTAAACATGCAAAGTCTGGGGTAGGTTATGAAAAACACTTCAATGCATTGAAATATCTCTTCAAATTTCATGACCATTTTGGTATCCACTTAAGTGGTGATGAATCATCTGCTGCGAAAGATCTTTTCGAAAATCCACTTGTTTTACCTTTCTCACAGCCTGAGTTGATTGTTGCCAACTGTGGTAATGCTGCTACCACGACATTTGGTATAACTCCTGCTGTACCTCATGGCTTTGGAATAGGGTACATCATTAAAGATGATCAAGTAGATTTGACCGTGACATCGCAATTTCGACAGGGTGACAGGTTAATGTTTATGTTAAGTTGGGTGTTAGGCGAAATTCGTTCCTACTGGAGGATGTCACGCGGCACTTCTCATAATAAAACTGGCGTGAAGATCAGTCCAGTGGTCGATAAATTGTATGAAATGGACAATGCAGTCAATAATCCTCCGAAACGTAACGGCCATACAGTCAATGGCTCCCGCAAAACATCTTCTTCATCACAGGTAAATTTGAACAGGTATGGCGGGTTTTTCGATTTGGAAGGCCACATTGACAGTAGGAATATATCTAAAACACCATCGATGAAGAATTTGCAGAAAACCTTCAATGGCTTGACCATGAGCGCAGACAATGATCATTCAAGTTCCGCAGTTTCCGTTCCCACAGAAAAGGAAAAATTAAATACAGGCCATGAAATTTTACAGATCCAACCGCGGGAAGTGGCAAGTAATGGGTTGGAAGCAGATGATGAGACAGATATTGAAATAGTTGCTGGCAATGCTGATGGCACATCCTCTTCGGCATCTTCAGCTACATCTCTAAATTCCAAGAAACGTAACGTCATCAACTCAAGATTCGATATTGATTTTGACCGCAGTCGTGTGGGTAGAAAGGTGGCGACCTTAGATCAATAAGAACGCTCTTTGTTTATCTATTTATTACTAGCATTATGCGTAAGCTTGGCGTGATGTGATATATACTTGTTATATGAAGTATATCAATATGCTTTAATTTATGCTTATTATTTGCTCTCATGTCAGTAACCCGGACCAGATTTGCCATTGAAAAAATTTTCATAGAATAGCCCGATGCTGATCATCAGTACAAAAGACAGAGATTTCATCTCGACCTGTACCAGGAGCTAAGCAAAGAGGGCGGAAAGAGCGTAATACACCGTTAACATCGCGCATTAGAGGTAGACCTAGCGTGTCCTCGCATAGTTCTTAGATTGTCGCTACGGCATATACGATCCGTGAGACGTAATTCCTGCAGCCCGGGGGATCCACTAGTTC |
| *pADH1-* *YAT2^C173G^-tYAT2* | CTATAGGGCGAATTGGGTACCGGGCCCCCCCCGACGAGATGCTCAGACTATGTGTTCTACCTGCTTGGACATCTTCGCGTATATGACGGCCCCCGGGAAGGTGAGACGCGCATAACCGCTAGAGTACTTTGAAGAGGAAACAGCAATAGGGTTGCTACCAGTATAAATAGACAGGTACATACAACACTGGAAATGGTTGTCTGTTTGAGTACGCTTTCAATTCATTTGGGTGTGCACTTTATTATGTTACAATATGGAAGGGAACTTTACACTTCTCCTATGCACATATATTAATTAAAGTCCAATGCTAGTAGAGAAGGGGGGTAACACCCCTCCGCGCTCTTTTCCGATTTTTTTCTAAACCGTGGAATATTTCGGATATCCTTTTGTTGTTTCCGGGTGTACAATATGGACTTCCTCTTTTCTGGCAACCAAACCCATACATCGGGATTCCTATAATACCTTCGTTGGTCTCCCTAACATGTAGGTGGCGGAGGGGAGATATACAATAGAACAGATACCAGACAAGACATAATGGGCTAAACAAGACTACACCAATTACACTGCCTCATTGATGGTGGTACATAACGAACTAATACTGTAGCCCTAGACTTGATAGCCATCATCATATCGAAGTTTCACTACCCTTTTTCCATTTGCCATCTATTGAAGTAATAATAGGCGCATGCAACTTCTTTTCTTTTTTTTTCTTTTCTCTCTCCCCCGTTGTTGTCTCACCATATCCGCAATGACAAAAAAATGATGGAAGACACTAAAGGAAAAAATTAACGACAAAGACAGCACCAACAGATGTCGTTGTTCCAGAGCTGATGAGGGGTATCTCGAAGCACACGAAACTTTTTCCTTCCTTCATTCACGCACACTACTCTCTAATGAGCAACGGTATACGGCCTTCCTTCCAGTTACTTGAATTTGAAATAAAAAAAAGTTTGCTGTCTTGCTATCAAGTATAAATAGACCTGCAATTATTAATCTTTTGTTTCCTCGTCATTGTTCTCGTTCCCTTTCTTCCTTGTTTCTTTTTCTGCACAATATTTCAAGCTATACCAAGCATACAATCAACTATCTCATATACAATGTCAAGCGGCAGTACTATTGTTTCGTCTGACAAGTCGGAGCGAACATTTAAGCATGAGGAAGAACTACCCAAGTTACCATTACCTAAACTCCGTGACACTTTGCAGCGTCTGAAGGAGAGCTTAGAGCCATTGTATTATGCTGATGGTTACTATCAACATCCTTTAGATCGAGAACAAATTGAGAAGCTGTCATCCATAATAAGAGATTTTGAAGAGAATCCCGTAAGTGAGAAGCTGCAATCTAAACTACAGAGCTACCATGACACTAGGGATTGTTACCTTGATGAATTACACTTAGACATCAATAACCAGACCTCTACTAGGGAGATTCAGGACGATGTCCTACCCAGGAATCCCTTCTTGGTTCTCGCAGATGACGCTTTGCCAAACATCACTCAAGCTGATAGGTCAGCCGTCCTAGTGCACTCTGCGGCCAGGTTTATTTCTGCTTTGAAACAAGATTTGTTGCCACCAGATATCAATGCCACCAATGGCAAACCACTGTCAATGGCTCCATTTCTGAACTTGTTCGGTACTACTCGTAGTCCTGTTTTTCAGCGCGGTGAGGTTGAAAATTTTGATTTGAACAAACCTTACACAGCATCTGATTTAGAAGACCCTGATTATTCAAGCGATGAGGGCGATAACGACGAACCCACCCAAAAAGACTTTGATGACCGTAAAAGAAAACACGAAGAAGACATATTCACTGGCAATGGTATAACTATAAAAAGACATCCGGATAGCAAACACATCTTGATCATCTCCAGGGGCCAATACTATACACTGGAAGTACTTGATTCGACGAATAAAATTATATACACTGCTGCAGAATTAACCACAATTTTCAACCATATAATAAAAGATTCTTCTGGCATTGAAAAATCAACTGCGTTAGGTAGTTTGACCTCTCACTCTTTTAGAAACTGGAAATATGCAAGGAAGAGACTACAGAAAAGGTATCCTAATGAATTACATCGCATAGACTCTGCTCTGTTCGTGTTAGTGCTGGACGAGTCACAAGAAGAAACCACCAATGATGGTGATGATACCGCTGATATCAGTCAAATGTTTAACAGGACCATCACTGAGCGAGACAAGAAATGTACTTCCGCCAATTGTAAGAGAGTGTTTTACGGTACTTCTATAATAAATAGCAAAGGCCATCAAGTGGGTTCATGTGTTTCTCGTTGGTATGATAAATTACAGTTAGTTGTCACTGCAGATGCAAAAGCCACAGTGATTTGGGATTCCTTCACATGTGACGGGTCAGTCGTGCTTCGGTTTACTTCTGAAATTTATACAGAATCAGTCTTGAGATTAGCTAGAGACGTTAACGCCGGTGACCCACAGTTTTCACTCTGGCCTAACGTGACACAAATGGATCCTGAAACTAAAAAACTGATGACGGCGACCATAAGTGCCGACGGCGGAGGCCCTTCTGAAATTGACCCTAAGTTGGTTGTAAATAAAATCGATTGGTCTTTCAGTAACATCCTGAACACACATGTCCATTTGTCAGAAACCAAACTAGCTGATCTGATTTCCAAGTATGATATTGTTCGCGCTTCTATTCCATTGGGCAGAAGGTCAGCTCAAAGATTAGGAGTCAAGCCCGATTCGATGGTTCAAGTAGCTTTACAAATAGCTCACTATGCTTTGTATGGAAGAATGGTGTTTGGATTAGAACCAGTATCCACACGTGGTTTCAAAAACTCGAGATCATCTTTTATTAACATTCAAAGTCAAGCGCTGTTGGAACTATGTCAATTGTTTATTTCTAGCTCCATTGATGGGACAGATAAATTAGACAAATTTATCCAAACCTGCGAAACACATAATAACATGGTTAAACATGCAAAGTCTGGGGTAGGTTATGAAAAACACTTCAATGCATTGAAATATCTCTTCAAATTTCATGACCATTTTGGTATCCACTTAAGTGGTGATGAATCATCTGCTGCGAAAGATCTTTTCGAAAATCCACTTGTTTTACCTTTCTCACAGCCTGAGTTGATTGTTGCCAACTGTGGTAATGCTGCTACCACGACATTTGGTATAACTCCTGCTGTACCTCATGGCTTTGGAATAGGGTACATCATTAAAGATGATCAAGTAGATTTGACCGTGACATCGCAATTTCGACAGGGTGACAGGTTAATGTTTATGTTAAGTTGGGTGTTAGGCGAAATTCGTTCCTACTGGAGGATGTCACGCGGCACTTCTCATAATAAAACTGGCGTGAAGATCAGTCCAGTGGTCGATAAATTGTATGAAATGGACAATGCAGTCAATAATCCTCCGAAACGTAACGGCCATACAGTCAATGGCTCCCGCAAAACATCTTCTTCATCACAGGTAAATTTGAACAGGTATGGCGGGTTTTTCGATTTGGAAGGCCACATTGACAGTAGGAATATATCTAAAACACCATCGATGAAGAATTTGCAGAAAACCTTCAATGGCTTGACCATGAGCGCAGACAATGATCATTCAAGTTCCGCAGTTTCCGTTCCCACAGAAAAGGAAAAATTAAATACAGGCCATGAAATTTTACAGATCCAACCGCGGGAAGTGGCAAGTAATGGGTTGGAAGCAGATGATGAGACAGATATTGAAATAGTTGCTGGCAATGCTGATGGCACATCCTCTTCGGCATCTTCAGCTACATCTCTAAATTCCAAGAAACGTAACGTCATCAACTCAAGATTCGATATTGATTTTGACCGCAGTCGTGTGGGTAGAAAGGTGGCGACCTTAGATCAATAAGAACGCTCTTTGTTTATCTATTTATTACTAGCATTATGCGTAAGCTTGGCGTGATGTGATATATACTTGTTATATGAAGTATATCAATATGCTTTAATTTATGCTTATTATTTGCTCTCATGTCAGTAACCCGGACCAGATTTGCCATTGAAAAAATTTTCATAGAATAGCCCGATGCTGATCATCAGTACAAAAGACAGAGATTTCATCTCGACCTGTACCAGGAGCTAAGCAAAGAGGGCGGAAAGAGCGTAATACACCGTTAACATCGCGCATTAGAGGTAGACCTAGCGTGTCCTCGCATAGTTCTTAGATTGTCGCTACGGCATATACGATCCGTGAGACGTAATTCCTGCAGCCCGGGGGATCCACTAGTTC |
| *pTDH3-CAT2-His_6_-tCYC1* | CTCGAGATAAAAAACACGCTTTTTCAGTTCGAGTTTATCATTATCAATACTGCCATTTCAAAGAATACGTAAATAATTAATAGTAGTGATTTTCCTAACTTTATTTAGTCAAAAAATTAGCCTTTTAATTCTGCTGTAACCCGTACATGCCCAAAATAGGGGGCGGGTTACACAGAATATATAACATCGTAGGTGTCTGGGTGAACAGTTTATTCCTGGCATCCACTAAATATAATGGAGCCCGCTTTTTAAGCTGGCATCCAGAAAAAAAAAGAATCCCAGCACCAAAATATTGTTTTCTTCACCAACCATCAGTTCATAGGTCCATTCTCTTAGCGCAACTACAGAGAACAGGGGCACAAACAGGCAAAAAACGGGCACAACCTCAATGGAGTGATGCAACCTGCCTGGAGTAAATGATGACACAAGGCAATTGACCCACGCATGTATCTATCTCATTTTCTTACACCTTCTATTACCTTCTGCTCTCTCTGATTTGGAAAAAGCTGAAAAAAAAGGTTGAAACCAGTTCCCTGAAATTATTCCCCTACTTGACTAATAAGTATATAAAGACGGTAGGTATTGATTGTAATTCTGTAAATCTATTTCTTAAACTTCTTAAATTCTACTTTTATAGTTAGTCTTTTTTTTAGTTTTAAAACACCAAGAACTTAGTTTCGAATAAACACACATAAACAAACAAAATGAGGATCTGTCATTCGAGAACTCTCTCAAACTTAAAGGATCTTCCGATAACGTCAAGGAGAGCAATGCATTCGGCCATTGTCAATTACTCCACCCAAAAGGCCCAATTTCCCGTAGAGACAAATAATGGGGAACACTATTGGGCGGAAAAGCCGAACAAATTCTACCAGAACAAAAGGCCCAATTTTCAAGGCATTACCTTTGCTAAACAACAAGACTTACCATCATTACCCGTGCCCGAATTGAAGTCTACACTTGACAAGTATTTGCAAACCATCCGCCCATTTTGCAATGATGTAGAAACTTTTGAAAGACAGCAGCTGTTATGTAAGGACTTCTCGGAGCACATGGGGCCTATCTTACAAGACCGATTGAAAGAGTATGCCAACGATAAAAGAAACTGGATGGCCAAGTTTTGGGATGAACAATCCTATTTACAATACAACGATCCTATTGTTCCATACGTCTCTTATTTTTATTCTCATATGCCATTACCGAATCATTTATCGAAGATCGATAATGATCCTTTGATTAAGGCTACTGCGATTATCTCAACCGTGGTTAAATTCATCGAAGCTATTAAAGATGAATCTTTACCCGTAGAAATTATCAAAGGTATGCCATTTTGTATGAATAGTTTTTCATTGATGTTTAACACTTCGAGATTGCCTGGTAAGCCAGAGGATAACCAAGATACAAATATTTTTTATTCAGTTTATGAGAACAACTTTGTAACTATCGCTTATAAAGGGAAGTTTTACAAACTGATGACCCATGACGGGAATGACAAACCGCTTTCCGAAAACGAAATCTGGAGGCAACTGTACTCTGTGGTATTCCAAGGATCGCAGTCCGATCCCAAACTAGGTGGCATTGGTTCTCTCACCTCTTTACCTCGTGATCAATGGCGTGAAGTACATATGGAGCTTATGAAGGATCCTATTTCTCAGGATTCACTAGAAACAATCCATAAGTCTTCCTTTATGCTATGTTTGGATCTTGACCAATCCCCTGTCACTTTGGAAGAAAAGTCAAGAAATTGCTGGCACGGTGATGGTATTAACAGATTCTACGATAAGTCTTTACAGTTCCTAGTCACCGGTAATGGTTCATCAGGTTTCTTAGCTGAACACTCGAAGATGGATGGTACGCCAACATTGTTTTTAAATAACTACGTTTGTCAGCAGTTGAATAAACTAGATGTGGATGACTTCATGAGAAAAGTAATTACGCCATCATCTACGGTGGCAATGAAACCTATGGAACTGCCCTTCATTATCACACCGAAGATTCATAAAGCAATCGAATCTGCCCAACTACAATTTAAGGAAACAATTGGTGAGCATGACCTACGTGTTTGGCACTACAACAAATATGGAAAAACGTTTATAAAACGCCATGGCATGTCACCTGATGCATTTATTCAACAAGTTATCCAACTGGCGGTTTTCAAATATCTGAAACGACAACTACCAACTTACGAGGCTGCTTCCACGAGAAAATACTTCAAAGGCCGTACTGAAACTGGTAGATCTGTGTCCACCGCCTCCTTAGAATTTGTTTCTAAATGGCAAAATGGCGATGTTCCTATTGCAGAAAAGATTCAGGCTTTGAAACATTCTGCAAAAGAGCATTCGACGTACCTGAAAAATGCTGCAAATGGTAATGGTGTCGATCGTCATTTCTTCGGTCTAAAGAATATGCTAAAATCTAATGATGACCAAATTCCGCCCCTTTTCAAAGATCCCTTATTTAATTATTCTTCAACTTGGTTGATCTCCACATCTCAACTATCTTCGGAATATTTTGACGGTTATGGTTGGTCCCAAGTAAATGACAACGGGTTTGGACTGGCATACATGTTGAATAACGAGTGGCTGCATATCAATATTGTCAACAAACCAGCCAAGAGTGGAGCCAGTGTTAACAGATTACACTATTATTTATCTCAAGCTGCTGATGAAATTTTTGACGCCTTGGAAAATGAGAATAAACGAAAAGCAAAGTTACATCATCACCACCATCACTGACAGGCCCCTTTTCCTTTGTCGATATCATGTAATTAGTTATGTCACGCTTACATTCACGCCCTCCTCCCACATCCGCTCTAACCGAAAAGGAAGGAGTTAGACAACCTGAAGTCTAGGTCCCTATTTATTTTTTTTAATAGTTATGTTAGTATTAAGAACGTTATTTATATTTCAAATTTTTCTTTTTTTTCTGTACAAACGCGTGTACGCATGTAACATTATACTGAAAACCTTGCTTGAGAAGGTTTTGGGACGCTCGAAGGCTTTAATTTGCACTAGT |
